# Cochlear implantation in unilateral hearing loss: impact of short- to medium-term auditory deprivation

**DOI:** 10.3389/fnins.2023.1247269

**Published:** 2023-10-09

**Authors:** Mohammed N. Ullah, Ashley Cevallos, Sarek Shen, Courtney Carver, Rachel Dunham, Dawn Marsiglia, Jennifer Yeagle, Charles C. Della Santina, Steve Bowditch, Daniel Q. Sun

**Affiliations:** ^1^Johns Hopkins Medicine, Johns Hopkins University, Baltimore, MD, United States; ^2^Department of Otolaryngology – Head and Neck Surgery and Cochlear Implant Center, The Johns Hopkins Hospital, Johns Hopkins Medicine, Baltimore, MD, United States; ^3^Department of Otolaryngology – Head and Neck Surgery, University of Cincinnati College of Medicine, Cincinnati, OH, United States

**Keywords:** cochlear implant, single-sided deafness, speech perception, spatial hearing, auditory deprivation, health utility

## Abstract

**Introduction:**

Single sided deafness (SSD) results in profound cortical reorganization that presents clinically with a significant impact on sound localization and speech comprehension. Cochlear implantation (CI) has been approved for two manufacturers’ devices in the United States to restore bilateral function in SSD patients with up to 10 years of auditory deprivation. However, there is great variability in auditory performance and it remains unclear how auditory deprivation affects CI benefits within this 10-year window. This prospective study explores how measured auditory performance relates to real-world experience and device use in a cohort of SSD-CI subjects who have between 0 and 10 years of auditory deprivation.

**Methods:**

Subjects were assessed before implantation and 3-, 6-, and 12-months post-CI activation via Consonant-Nucleus-Consonant (CNC) word recognition and Arizona Biomedical Institute (AzBio) sentence recognition in varying spatial speech and noise presentations that simulate head shadow, squelch, and summation effects (S_0_N_0_, S_SSD_N_NH_, S_NH_N_SSD_; 0 = front, SSD = impacted ear, NH = normal hearing ear). Patient-centered assessments were performed using Tinnitus Handicap Inventory (THI), Spatial Hearing Questionnaire (SHQ), and Health Utility Index Mark 3 (HUI3). Device use data was acquired from manufacturer software. Further subgroup analysis was performed on data stratified by <5 years and 5–10 years duration of deafness.

**Results:**

In the SSD ear, median (IQR) CNC word scores pre-implant and at 3-, 6-, and 12-months post-implant were 0% (0–0%), 24% (8–44%), 28% (4–44%), and 18% (7–33%), respectively. At 6 months post-activation, AzBio scores in S_0_N_0_ and S_SSD_N_NH_ configurations (*n* = 25) demonstrated statistically significant increases in performance by 5% (*p* = 0.03) and 20% (*p* = 0.005), respectively. The median HUI3 score was 0.56 pre-implant, lower than scores for common conditions such as anxiety (0.68) and diabetes (0.77), and comparable to stroke (0.58). Scores improved to 0.83 (0.71–0.91) by 3  months post-activation. These audiologic and subjective benefits were observed even in patients with longer durations of deafness.

**Discussion:**

By merging CI-associated changes in objective and patient-centered measures of auditory function, our findings implicate central mechanisms of auditory compensation and adaptation critical in auditory performance after SSD-CI and quantify the extent to which they affect the real-world experience reported by individuals.

## Introduction

Single-sided deafness (SSD) refers to profound unilateral sensorineural hearing loss, in which patients exhibit significantly poorer hearing thresholds in one ear with normal or near-normal hearing in the contralateral ear (Snapp and Ausili, 2020). Loss of access to binaural hearing cues including interaural timing differences (ITDs) and interaural level differences (ILDs) leads to deficits associated with the head shadow effect (HSE), squelch, and summation (Bakal et al., 2021). The HSE refers to the ability of the head to serve as an acoustic barrier as it blocks sound waves traveling toward the contralateral ear relative to the source of the sound. Squelch results from the central processing of different amplitudes, frequencies, and timing as sound reaches each ear. Lastly, summation amplifies the perception of sound as an auditory stimulus reaches both ears (Mertens et al., 2015). Loss of these binaural cues is associated with changes in central auditory processing that together clinically manifest as impairments in speech discrimination, hearing in noise, and sound localization in SSD patients (Tillein et al., 2016; Vannson et al., 2020).

Cochlear implantation has emerged as an important tool in the clinical treatment of SSD, with demonstrated benefit for hearing, tinnitus, and quality of life (Van de Heyning et al., 2008; Arndt et al., 2017; Daher et al., 2023). In contrast to traditional strategies such as Contralateral Routing of Signal (CROS) or Osseointegrated Hearing Aids (OHA), a cochlear implant (CI) is the only device able to restore input to the deafened ear, and therefore provide some degree of bilateral function (Ericson et al., 1988; Hagr, 2007). Consequently, multiple studies have demonstrated improved hearing in noise and sound localization in SSD CI subjects (Firszt et al., 2012; Hansen et al., 2013; Litovsky et al., 2019; Speck et al., 2021). Additional improvements to tinnitus and quality of life have also been observed, though quality of life assessment methods in the literature are highly variable (Benítez et al., 2021; Lindquist et al., 2023). There is also a paucity of data that connects auditory assessments to the experience of SSD CI subjects, which represents the cumulative effects of central auditory mechanisms of compensation and adaptation, and integration of CI input, in real-world settings. Such patient-centered measures offer valuable insight into the real-world impact of SSD and associated CI use, as well as an emerging focus in biomedicine research (Tyler et al., 2009).

Due to atrophy of spiral ganglion neurons and cortical reorganization that occur with longer durations of deafness, the length of auditory deprivation prior to CI can have important impacts on auditory outcomes achieved after CI. This has been extensively studied in subjects with bilateral hearing loss (Shibata et al., 2011; Anderson et al., 2017; Grégoire et al., 2022). SSD differs from bilateral hearing loss due to intact acoustic input from the normal hearing ear that shapes cortical reorganization after onset of deafness as well as bimodal integration after CI (Han et al., 2021; Karoui et al., 2023). Clinically, a duration of deafness of 10 years is commonly considered the threshold at which individuals are less likely to benefit from CI, although some patients have obtained benefit with even longer durations of auditory deprivation (Muigg et al., 2020; Bernhard et al., 2021; Nassiri et al., 2022). United States FDA criteria for CI in SSD also limit patients to less than 10 years duration of deafness. However, spiral ganglion neuron atrophy and changes in central auditory processes occur continuously after onset of deafness, and the extent to which length of auditory deprivation can change auditory performance with CI for subjects within the current clinical window of 10-year duration of deafness is unclear (Nassiri et al., 2022).

Due to small number of subjects, limited scopes of assessment, and heterogeneity in both the audiologic and quality-of-life assessments performed, CI-associated audiologic outcomes and impact to quality of life for SSD subjects remain incompletely understood. Additionally, as CI benefit is highly variable between individuals, more research exploring factors that may impact CI outcomes, such as duration of deafness, is required. We present data from a prospective observational study of a population of SSD subjects with varying durations of auditory deprivation before and after CI. We hypothesize improvements in performance across audiologic and patient-centered measures following implantation. Additionally, we hypothesize a correlation between the duration of deafness and outcomes (both audiometric and patient-reported) in our SSD-CI patient population. By linking data from audiological testing, patient-centered quality of life measures, and real-world device use, this study investigates how auditory input provided by the CI translates into real-world experience for subjects, and the impact of short (<5 years) to medium (5–10 years) durations of deafness, with findings relevant for further investigations of neuroscientific mechanisms that underlie compensation and adaptation after SSD and bimodal integration after CI.

## Methods

### Patient recruitment

This was a prospective cohort observational study (NCT # 05052944) of patients undergoing CI for SSD recruited from the Johns Hopkins Cochlear Implant Center between January 2020 and August 2022 on a rolling basis. Data analysis was performed in January 2023. Inclusion criteria for study participants were patients diagnosed with SSD who met FDA candidacy criteria for CI in SSD (Supplementary Table 1), including audiometric pure tone average (PTA) of >80 dB HL at 500, 1000, 2000, and 4,000 Hz in the deafened ear and ≤ 30 dB HL in the contralateral ear, score of ≤5% on a developmentally-appropriate monosyllabic word list, and at least 1-month trial of a CROS hearing aid without subjective benefit. Patients who failed to meet these criteria, declined consent for data collection, were unable to perform audiologic tasks (e.g., non-English speaking patients) or were medically or surgically contraindicated for CI surgery were excluded from the study. This study was approved by the Johns Hopkins School of Medicine Institutional Review Board (IRB00230644), and informed consent was obtained prior to enrollment.

### Data collection

Clinical and demographic data were compiled in a study database. Duration of deafness was extracted from medical charts. Speech perception performance of the implanted ear and patient-centered outcome measures were performed at pre-implant evaluation, and 3-, 6-, and 12-months following CI activation. At each time point, audiologic function of the implanted ear was evaluated using consonant-nucleus-consonant (CNC) words and Arizona Biomedical Institute (AzBio) sentence tests by an experienced CI audiologist during routine clinic visits. Word and sentence lists were chosen at random from prespecified sets at each visit. CNC word recognition in the implanted ear was assessed in sound field at 60dBSPL. The candidate ear was appropriately fit with a power hearing aid programmed to prescriptive formula, NAL-NL2, for the unaided air conduction thresholds. The non-implanted ear was masked using speech noise presented via insert headphone at a signal to noise ratio of +4 dB. AzBio sentence recognition was performed with signal presented at 60dBSPL with +8- or + 5-dB signal-to-noise ratio multi-talker babble in three main spatial presentations: (1) speech and noise projected from in front of the participant (S_0_N_0_), (2) speech projected toward the participant’s candidate (CI) SSD ear and noise projected toward their non-candidate (non-CI) ear with normal hearing (S_SSD_N_NH_), and (3) speech projected toward the study participant’s non-CI ear and noise projected to their CI ear (S_NH_N_SSD_; NH = Normal Hearing).

Patient-centered assessments were collected via the following survey instruments: Tinnitus Handicap Inventory (THI), Spatial Hearing Questionnaire (SHQ), and Health Utility Index Mark 3 (HUI3). These instruments were selected to evaluate real-world experience of CI subjects. The THI quantifies the self-reported severity of tinnitus on one’s quality of life through a 25-question survey, to which subjects can answer with “yes,” “sometimes,” or “no.” Each answer choice is weighted differently and totaled to provide a THI score on a scale from 0 to 100 quantifying the patient’s tinnitus severity. Classification of THI scores is as follows: 0–16 indicates slight to no handicap; 18–36 mild handicap, 38–56 moderate handicap; 58–76 severe handicap; and 78–100 catastrophic handicap (Wakabayashi et al., 2020).

The SHQ is a patient self-report tool designed to assess patient experience in various spatial hearing scenarios. It consists of 24 items, each a different spatial hearing scenario scored on a scale from 0 to 100, with lower scores denoting more difficulty with the scenario and therefore greater impairment. An aggregate score was derived from the average of all 24 items, which encompassed 8 sub-domains: perception of male, female, and children’s voices, music, source localization, understanding speech in quiet, understanding speech in noise with speech and noise projected from in front of the individual, and understanding speech in noise with speech and noise presented in separate ears.

The HUI3 is a multi-attribute health-status assessment quantifying an individual’s health-related quality of life with respect to 8 dimensions: vision, hearing, speech, ambulation, dexterity, pain, emotion, and cognition. The responses offer 3–6 levels of discrimination, with results combined formulaically in line with single- and multi-attribute utility scoring systems on a scale from −0.36 to 1.00, where 1.00 represents perfect health and 0 represents death (HUI3 scoring allows for health scores representing states worse than death; Horsman et al., 2003). HUI3 has been validated at the population level across multiple common disease conditions allowing comparative analysis of health utility (Grootendorst et al., 2000; Horsman et al., 2003; Maddigan et al., 2006; Kaplan et al., 2007; Asakawa et al., 2008; Guertin et al., 2018).

Device use was monitored using the CI manufacturer’s proprietary data-logging system. Device use was reported in hours/day of wearing time. At each visit, the hours/day of use for the previous time interval was recorded. For example, the device use measurement at the 6 month time interval was representative of the previous 3 months (time from the 3 month visit to the 6 month visit).

### Statistical analysis

For this rolling, open recruitment study, statistical significance of differences in measures between study time points was calculated using univariate linear mixed effects regression models with subject ID’s entered as random effects to address missing data, age as a covariate, and measured variables as fixed effects. These models were then analyzed via *t*-tests using Satterthwaite’s method for statistical significance, which was set at *p* < 0.05. Further subgroup analysis was performed according to the period of deafness from the onset of a patient’s SSD symptoms to the patient’s CI surgery, referred to hereafter as the duration of deafness. Patients were stratified into two groups: <5 years and 5–10 years duration of deafness at time of implantation. The data were not normally distributed for this population, so median scores (IQR) are reported. All statistical analyses were performed using R Statistical Software (v4.2.3; R Core Team, 2021). Linear mixed-effects modeling was conducted via the lmerTest package (v3.1.3; Kuznetsova et al., 2017).

## Results

### Demographic and clinical data

**Figure 1:**
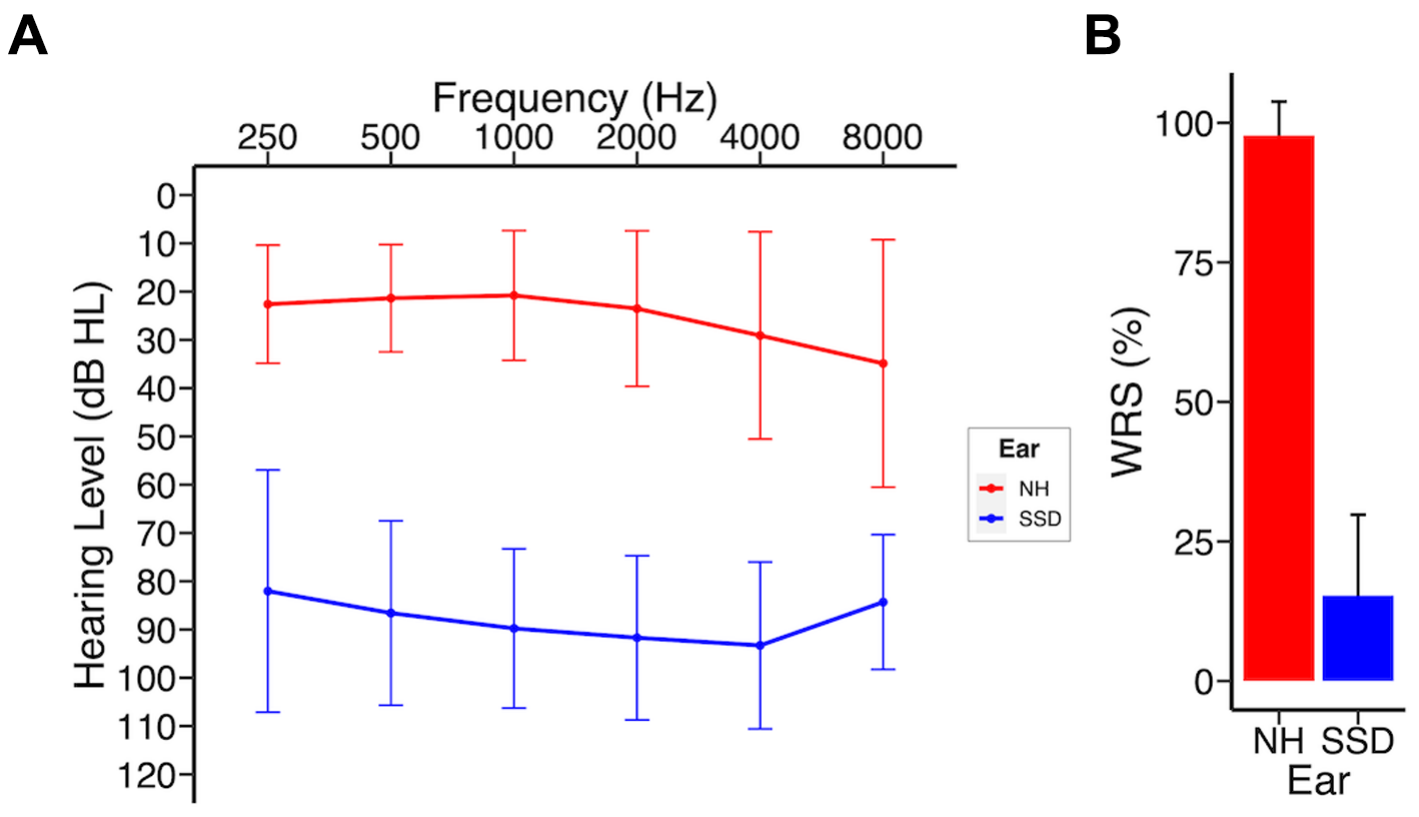
SSD patients at baseline; NH, normal hearing ear; SSD, impacted ear. **(A)** Baseline audiogram **(B)** Word recognition scores (NU-6). Data presented as mean ± 1 SD.

Demographic and clinical data of the study cohort are presented in Table 1. A total of 44 subjects who underwent CI implantation were enrolled at the time of this analysis and included 20 males (45.5%) and 24 females (54.5%), with a mean (SD) age of 51.9 (14.3) years at time of implantation. As a rolling study, 33 patients reached 3 months, 25 patients reached 6 months, and 16 patients reached 12 months at time of analysis. One patient was lost to follow up after 3 months due to relocation out of the study area. The most common etiology of SSD was idiopathic sudden sensorineural hearing loss in 27 patients (61%), including 22 patients with concurrent vertigo consistent with labyrinthitis. Other etiologies included Meniere’s disease (5 patients), iatrogenic causes (post-surgical, 4 patients), and schwannomas (2 patients; Supplementary Table 2). Duration of deafness at time of implantation was <5 years in 39 patients (88%) and 5–10 years in 5 patients (12%). The right ear was implanted in 19 (43%) of subjects. Audiological and patient reported outcomes did not differ by laterality of implant. MED-EL Flex 28 was implanted in 35 patients and Cochlear 632 in 9 patients. Nineteen patients underwent CI evaluation and met inclusion criteria but elected not to proceed with implantation. These subjects were not included at the time of this analysis. Analysis of this subgroup revealed no significant differences in baseline CNC, AzBio, THI, SHQ, or HUI3 scores compared to those who underwent CI (Supplementary Table 3). Survey response rates were 68, 64, 76, and 94% at pre-implant, 3 months, 6 months, and 12 months, respectively. No significant differences were observed in age, sex, race, pre-implant audiometric thresholds, or laterality of implant when stratified by duration of deafness. Analysis of duration of deafness as a continuous variable yielded significance when exploring its impact on pre-implant spatial hearing scores, but not at any other time point in this study (Supplementary Table 7). Figure 1 shows the composite audiogram of the study population prior to CI. Average word recognition scores (WRS) using NU-6 words in the normal and deafened ears of participants at their initial CI evaluation were approximately 98 and 14.3%, respectively (Figure 1).

**Table 1:** Summarized demographic and clinical characteristics of enrolled subjects.

Demographic	*N* = 44 subjects
Age at implantation, mean (SD), *y*	51.6 (14.0)
Patient sex	
Male	20 (45.5%)
Female	24 (54.5%)
Patient race	
White	35 (79.5%)
Black or African American	6 (13.6%)
American Indian or Alaskan native	1 (2.3%)
Hispanic or latino	2 (4.6%)
Laterality of implant	
Right	19 (43.2%)
Left	25 (56.8%)
Cochlear implant device	
MED-EL	35 (79.5%)
Cochlear Ltd	9 (20.5%)
Duration of deafness	
≤5 years	39 (88%)
5–10 years	5 (12%)

### Speech perception performance

**Figure 2:**
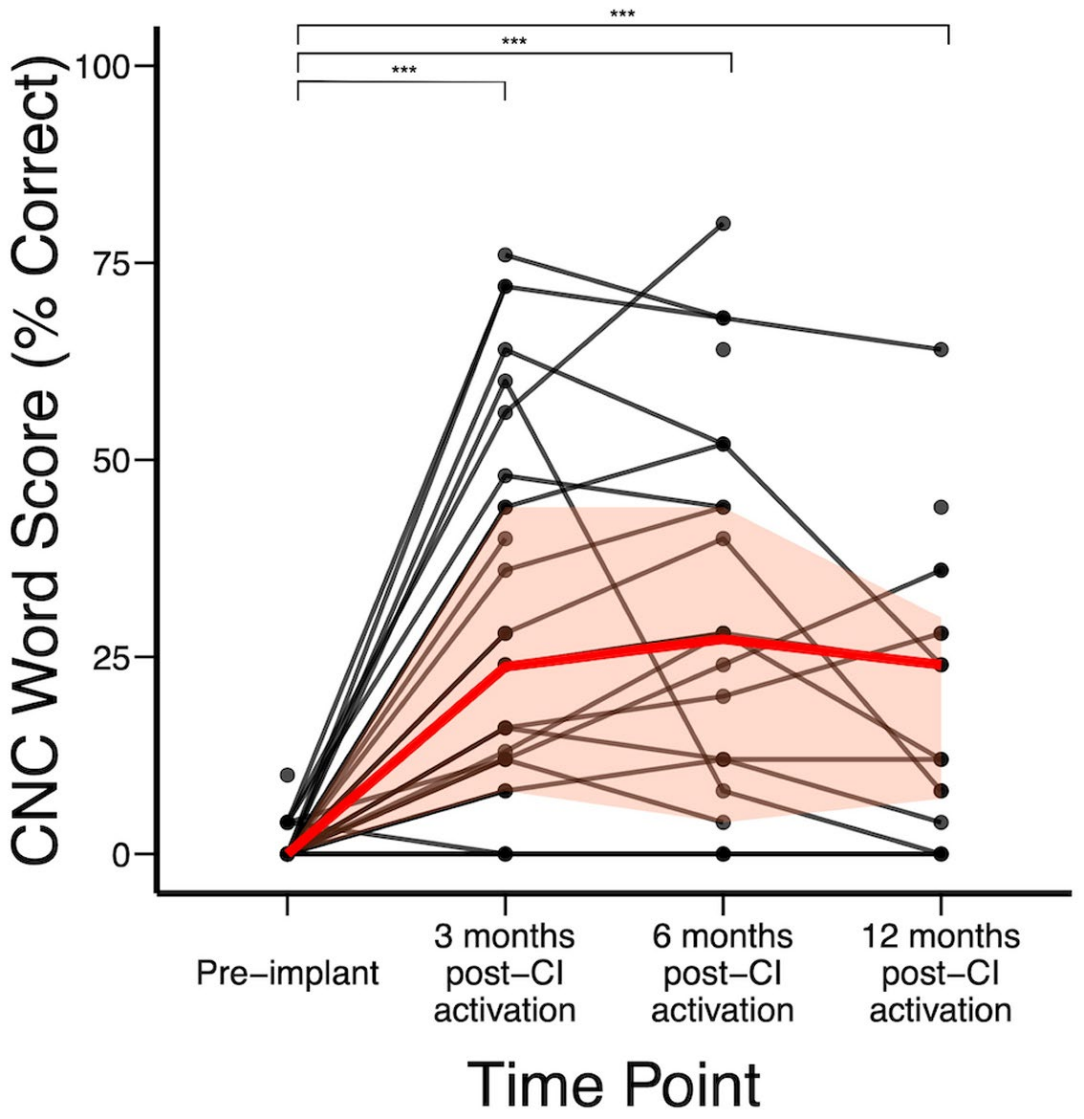
CNC word scores over time; red represents median (IQR) CNC word recognition scores over time. *p < 0.05; **p < 0.01; ***p < 0.001.

Figure 2 shows the audiologic trajectories of CI recipients. In the subject cohort analyzed, CNC word scores were available for 44 subjects pre-implant. Overall, significant improvement was achieved by 3 months post-CI (Figure 2). Median (IQR) CNC word scores pre-implant and at 3-, 6-, and 12-months post-CI activation were 0% (0–0%), 24% (8–44%), 28% (4–44%), and 18% (7–33%), respectively. Compared to baseline, statistically significant change in CNC score was achieved by 3 months (*p* < 0.0001) and further changes between 3-, 6-, and 12- months did not reach statistical significance. We did not detect a statistically significant difference at each time point when stratified by duration of deafness. However, not all individuals benefited equally from CI. Of 5 subjects who had no or minimal improvement in CNC after CI, subject A had hearing loss due to Charcot Marie Tooth, which negatively affects CI outcomes (Chaudhry et al., 2020; Kobayashi et al., 2021), subject B had no observed benefit to CNC scores but reported significant subjective improvement on SHQ and HUI questionnaires, subject C had 10-year duration of deafness prior to CI, and subjects D and E were non-users (declined to use and experienced difficulty with use due to employment gear, respectively). In addition, subjects F, G, and H had decline in hearing in the implanted ear between 3 and 12 months after initial improvement. Upon further clinical review, it was observed that cancer recurrence occurred in subject F that limited CI use, subject G developed tolerance issues to CI sound (hyperacusis), and etiology of hearing decline was unknown in subject H (Supplementary Table 4).

**Figure 3:**
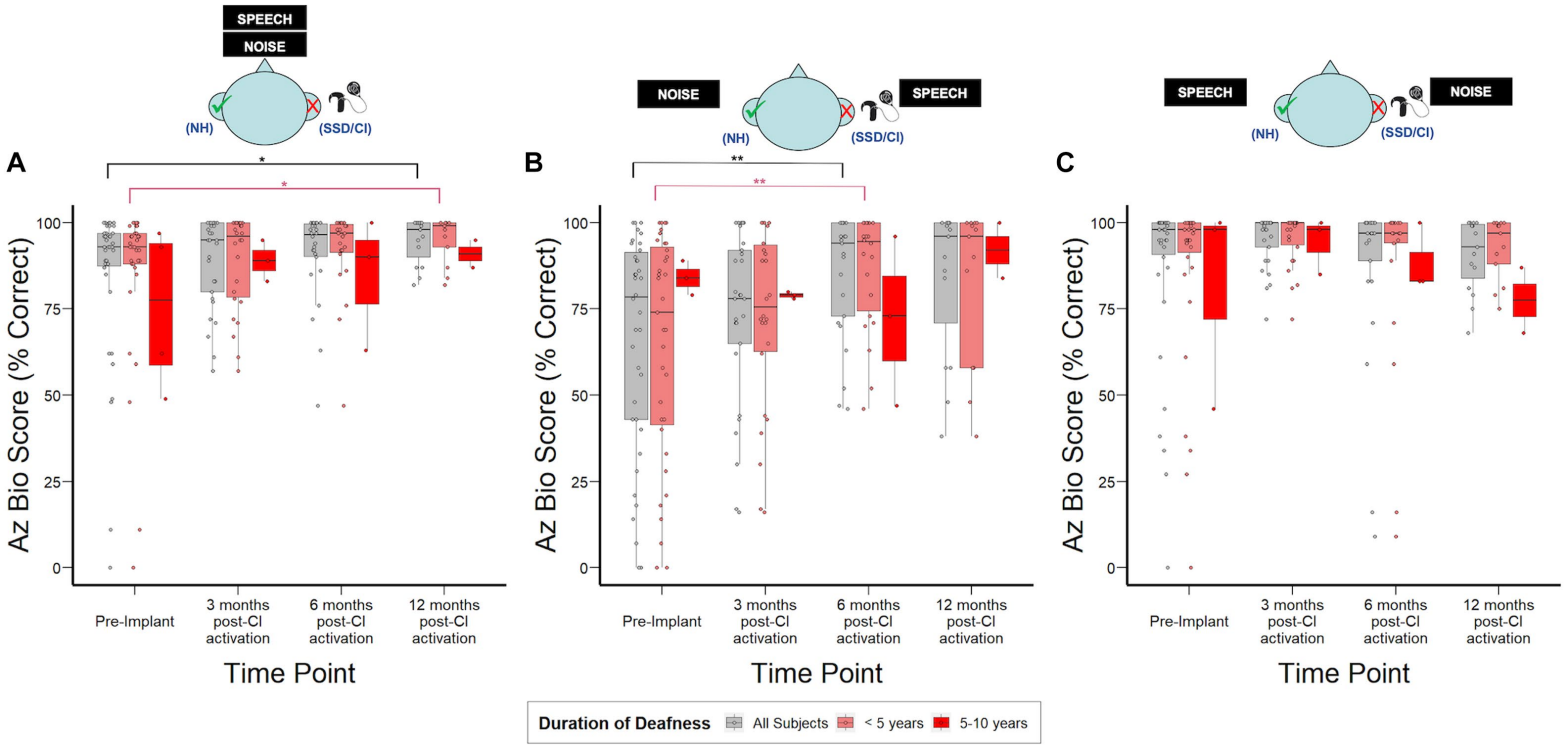
AzBio sentence recognition scores over time stratified by duration of deafness (<5 years, 5–10 years) prior to implantation. **(A)** S_0_N_0_: speech and noise projected from in front of the subject. **(B)** S_SSD_N_NH_: speech projected toward the SSD ear and noise toward the normal ear. **(C)** S_NH_N_SSD_: speech projected toward the normal ear and noise toward SSD ear; NH, normal hearing ear (green check); SSD, impacted ear (red “X”). *p < 0.05; **p < 0.01; ***p < 0.001.

Figure 3 shows hearing in noise assessment using AzBio in 3 distinct spatial configurations. CI recipients demonstrated the most benefit in the S_SSD_N_NH_ configuration, with a median improvement of 20% in AzBio scores relative to pre-implantation by 6 months post-CI activation (*p* = 0.005). In the S_0_N_0_ configuration, CI recipients demonstrated a modest but statistically significant 5% increase in AzBio scores by 12-months post-CI activation (*p* = 0.03). No significant changes were observed in the S_NH_N_SSD_ configuration. Additionally, no significant differences were observed in AzBio scores when stratified by duration of deafness.

### Patient-reported outcomes

#### Tinnitus

Pre-implant THI was completed by 30/44 (68%) subjects while post-implant THI was completed by 21 subjects at 3 months, 19 subjects at 6 months, and 15 subjects at 12 months post-CI activation. The largest significant reduction in THI scores was observed at 3 months-post CI activation, with median (IQR) scores dropping from 28 (8–48), indicating mild handicap due to tinnitus severity, to 12 (2–24), indicating slight to no handicap due to tinnitus severity (*p* < 0.001). This reduction subsequently plateaued and THI scores remained similar through 12 months post-CI activation (t_12_: 14 (6–22); *p* = 0.004). When stratified by duration of deafness, no statistically significant differences were observed in tinnitus severity (Supplementary Figure 1).

#### Quality of life – spatial hearing

**Figure 4:**
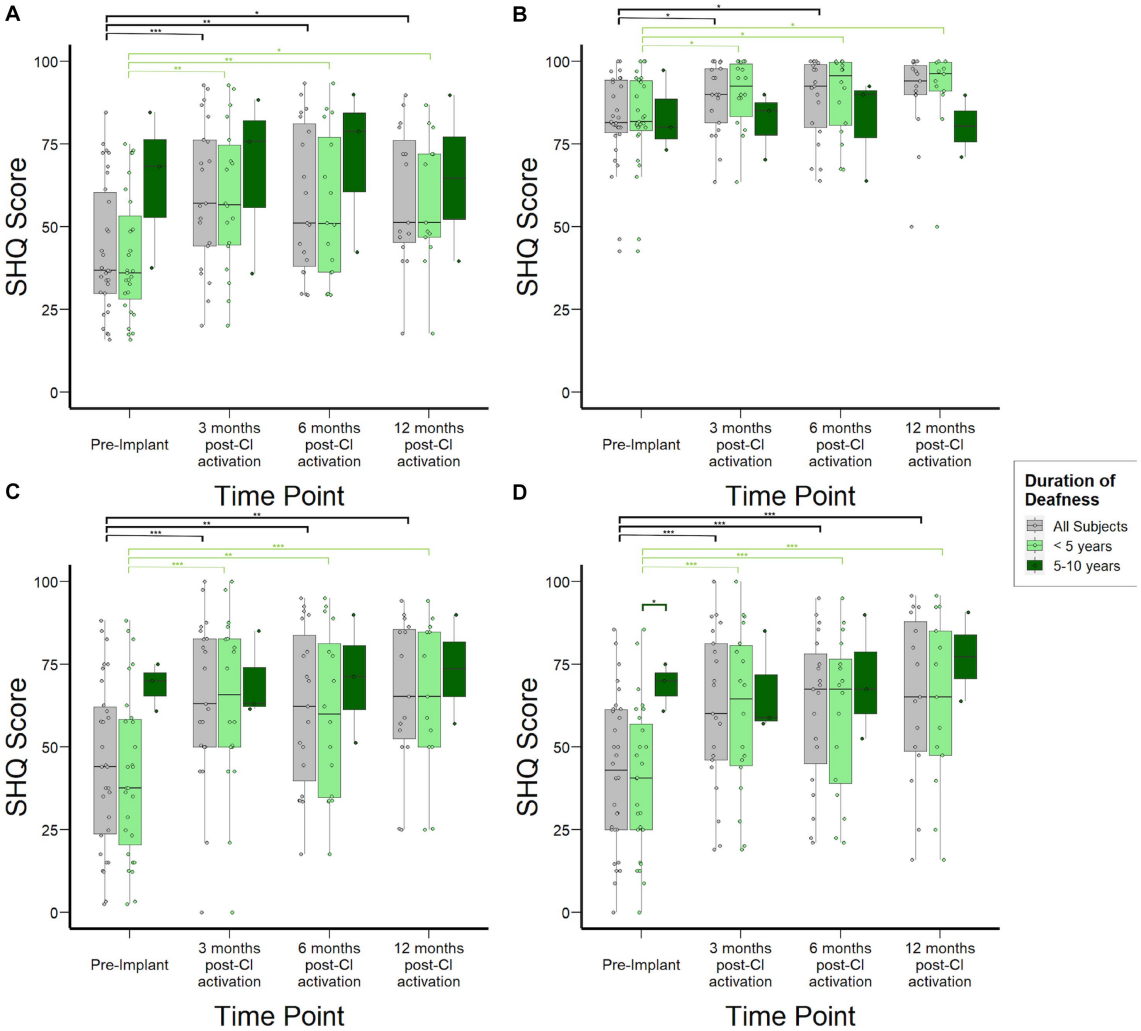
Spatial Hearing Questionnaire (SHQ) scores to assess subjective spatial hearing experiences over time stratified by duration of deafness (<5 years, 5–10 years) prior to implantation. **(A)** Overall aggregate SHQ scores. **(B)** SHQ scores in speech-in-quiet scenarios. **(C)** SHQ scores in scenarios where speech and noise are both projected from in front of the subject. **(D)** SHQ scores in scenarios where speech and noise are separated. *p < 0.05; **p < 0.01; ***p < 0.001.

Figure 4A shows overall SHQ scores for the study cohort at each time point and stratified by duration of deafness. The overall study population demonstrated improvement in spatial hearing-related quality of life, with statistically significant increases from 37 (30–60) at pre-implant to 57 (44–76) at 3 months (*p* < 0.01) and 51 (46–76) at 12 months post-CI activation (*p* = 0.03). These overall score improvements were driven by improvements in SHQ subdomains specifically related to spatial hearing in the context of male, female, and children’s voices as well as music. SHQ scores for speech in quiet in the overall population improved mildly at 6 months post-CI activation by 11 points (Figure 4B, *p* = 0.03). In contrast, subjects reported significant improvements in speech in noise scenarios both when speech and noise are presented in front (Figure 4C) and when they are presented from separate directions (Figure 4D). In both scenarios, the improvement manifested by 3 months post-activation (*p* < 0.001) and remained stable through 12 months post-activation. When examined by duration of SSD prior to CI, important differences emerged in the SHQ data. Subjects with 5–10 years duration of SSD reported better spatial hearing quality of life prior to implantation relative to those with SSD for <5 years. Although this difference did not reach statistical significance (*p* = 0.12), it was driven by improved scores in SHQ questions related to speech in noise scenarios (Figures 4C,D) and a statistically significant difference (*p* < 0.05) was reached specifically in scenarios where speech and noise are separated. Post-activation, subjects with 5–10 years duration of deafness reached SHQ scores similar to those with <5 years duration of SSD. Consequently, the magnitude of reported improvement in SHQ was reduced for those with 5–10 years duration of deafness at time of implantation.

#### Health utility

**Figure 5:**
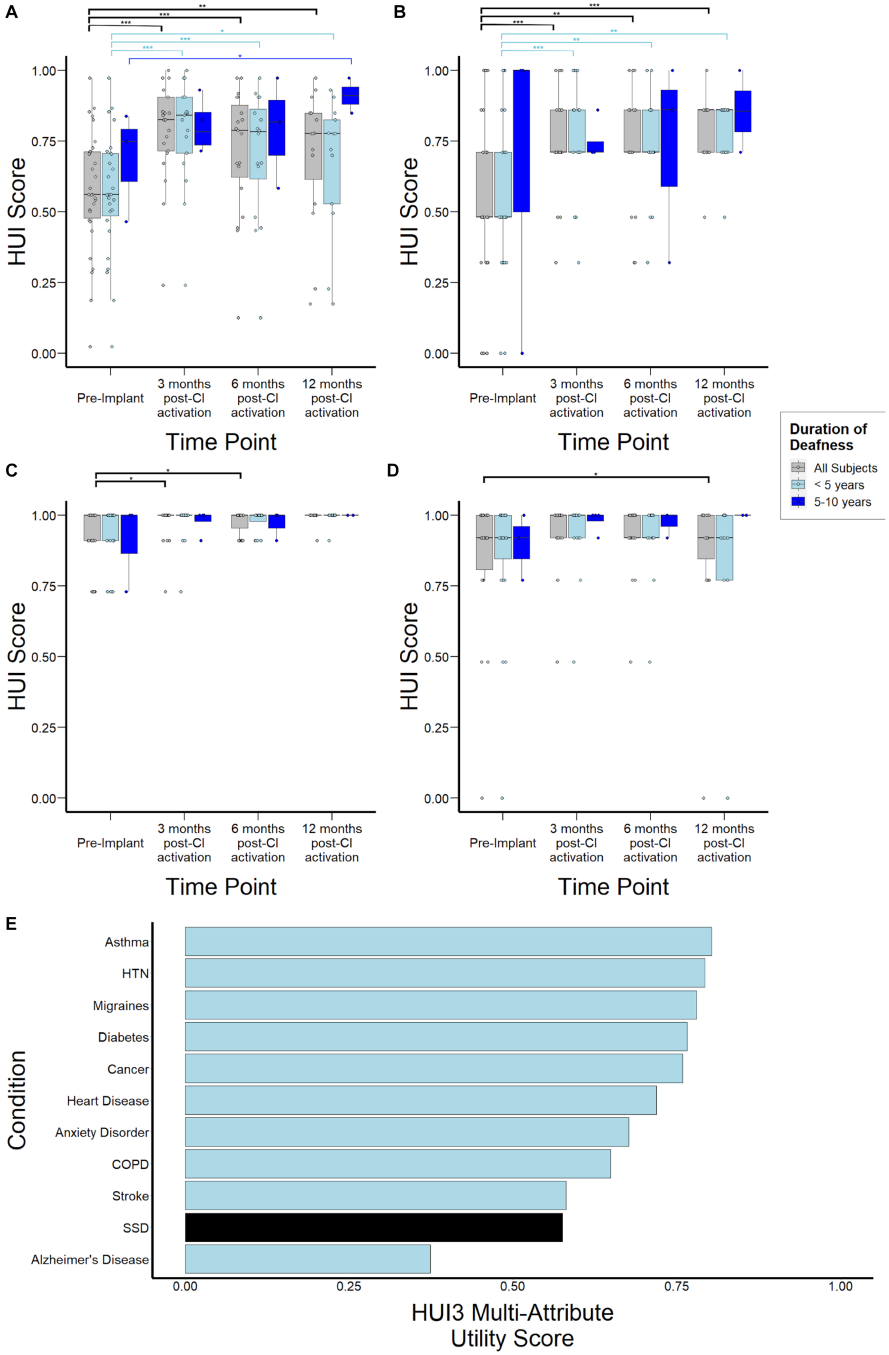
Health Utility Index Mark 3 (HUI3) scores to assess health-related quality of life over time stratified by duration of deafness (< 5 years, 5–10 years) prior to implantation. **(A)** Overall HUI3 multi-attribute utility scores **(B)** HUI3 single-attribute hearing scores **(C)** HUI3 single-attribute emotion utility scores **(D)** HUI3 single-attribute pain utility scores **(E)** HUI3 multi-attribute utility scores for major common debilitating conditions and at baseline for SSD patients. *p < 0.05; **p < 0.01; ***p < 0.001.

Median HUI3 scores were 0.56 (0.48–0.71) for the study population prior to CI, increased to 0.83 (0.72–0.91, *p* < 0.001) by 3 months post-activation, and remained stable at 0.79 (0.62–0.88, *p* = 0.82) and 0.78 (0.61–0.85, *p* = 0.62) at 6-, and 12-months, respectively (Figure 5A). When stratified by duration of SSD prior to CI, subjects with 5–10 years duration demonstrated lower HUI3 scores at 3- and 6-months follow-up, although this difference did not reach statistical significance (*p* = 0.67, *p* = 0.66). By 12-months post-activation, these subjects actually reported higher HUI3 scores than those with <5 years duration of SSD, though this difference also did not reach statistical significance (*p* = 0.08). Sub-domain analysis of single-attribute utility scores revealed that lower HUI3 scores in the overall study population prior to CI were primarily driven by poorer scores reported in the hearing subdomain (Figure 5B), and not by variance in emotional (Figure 3C) or pain (Figure 5D) subdomains that may be indirectly impacted in other health states. In particular, subjects with 5–10 years duration of deafness demonstrated higher variance in reported scores in the hearing subdomain at 6- and 12-months. Notably, when contextualized against other common health states, HUI3 scores for SSD were worse than anxiety (0.68), diabetes (0.77), COPD (0.65), and heart disease (0.72), and comparable to stroke (0.58; Figure 5E).

#### Device use

**Figure 6:**
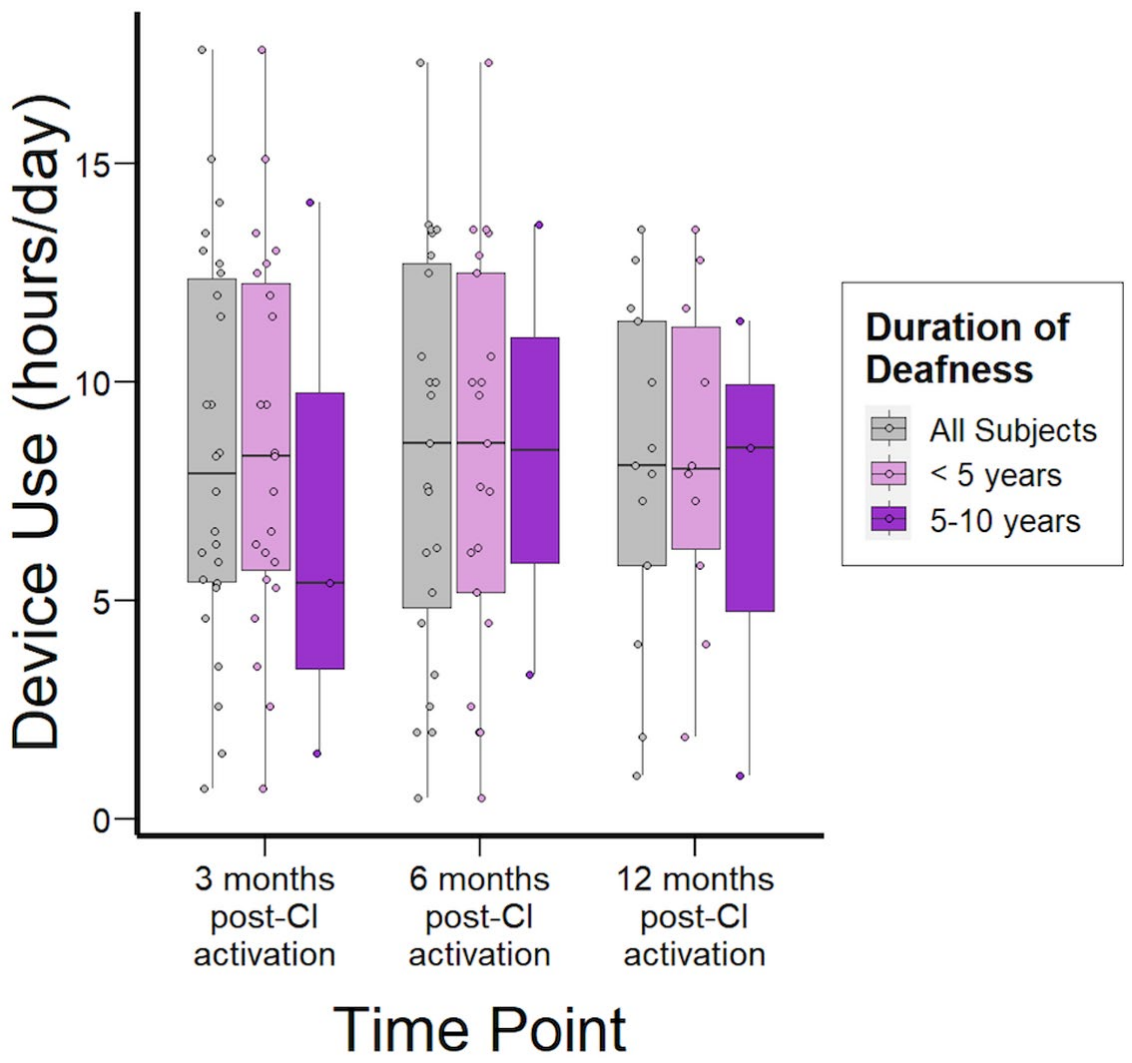
Device use data over time stratified by duration of deafness (<5 years, 5–10 years).

Device use analysis demonstrated consistent daily wear-time through 12 months following activation for the overall study population (Figure 6). When stratified by duration of deafness, those with 5–10 years demonstrated median (IQR) daily wear-time of 5.4 h/day (3.5–9.8) compared to 8.3 h (5.7–12.2) for the <5 years cohort (*p* = 0.82) at 3 months following activation. However, wear-time for the 5–10 years cohort steadily improved to a median (IQR) of 8.5 (6.2–11.3) hours daily and no significant difference was demonstrated at 6- and 12-months between groups.

## Discussion

In this study, we report data from a prospective observational study of a large cohort of adult subjects undergoing cochlear implantation after sustaining SSD. Importantly, our data show the pervasive and profound impact on health utility due to SSD (Figure 5), the significant overall improvement in auditory performance achieved after CI, and the real-world experience reported by subjects. Secondly, audiologic and patient-reported data are presented here within the scope of the current FDA criteria for CI in SSD, which allows for up to 10 years in duration of deafness, and suggest that overall, patients with longer periods of auditory deprivation exhibit similar improvement in outcomes post-CI compared to those with shorter periods. Although outcome differences did not reach statistical significance in our cohort, likely due to limited statistical power, emerging trends in these results are consistent with central processes that play an important role in mediating clinical outcomes in SSD.

### Speech understanding in quiet

Similar to previous studies on CI outcomes in SSD, speech understanding in quiet, as measured by CNC word scores, improved significantly in the deafened ear after implantation (Figure 2; Hansen et al., 2013; Sladen et al., 2017; Galvin et al., 2019; Sullivan et al., 2020). Interestingly, when compared to previous studies in subjects undergoing unilateral CI for bilateral hearing loss, SSD subjects demonstrated reduced plateau performance and earlier time to plateau, which has also been observed in other studies (Chang et al., 2010; Dillon et al., 2013; Holden et al., 2013). Whereas bilateral hearing loss patients typically reach plateau in 6–12 months and sometimes continue to improve over years, SSD patients in this cohort almost uniformly reached plateau at only 3 months post activation, consistent with other SSD CI studies (Cusumano et al., 2017; Buss et al., 2018). Simultaneously, SSD CI subjects demonstrate lower plateau scores relative to bilateral hearing loss patients, which may be related to challenges in isolating the CI ear during testing. Similar to other studies, CNC word scores in this study were obtained using sound field testing with masking noise presented via insert earphones to the normal-hearing ear. A signal to noise ratio of +4 dB was used to assess patients in the present study. While this SNR was selected to ensure complete isolation of the CI ear, the high level of noise may also lead to artifactually decreased CNC word scores in the implanted ear. Indeed, previous studies using lower noise levels (e.g., SNR +10 dB) have found higher CNC scores in SSD CI patients, although that strategy risks inadequate masking and confounding acoustic input from the normal hearing ear (Buss et al., 2018). Currently, there is a lack of specific audiometric standards for assessing speech recognition in SSD CI recipients. In children, when speech was directly streamed into the CI device, improved CNC word scores were observed (Park et al., 2021, 2023).

Alternatively, reduced speech recognition scores in SSD CI subjects may be related to cortical level reorganization in unilateral hearing loss (Bilecen et al., 2000; Ponton et al., 2001; Khosla et al., 2003; Burton et al., 2012). Patients with SSD exhibit greater neuronal activity in the auditory pathways associated with their normal hearing ear, suggesting the development of an aural dominance, or preference, for the normal ear (Li et al., 2006; Chang et al., 2020; Vannson et al., 2020). This has been thoroughly demonstrated in numerous animal studies. Studies in deaf cats in particular have simultaneously demonstrated a weaker representation of the deaf ear and a stronger representation of the normal hearing ear at the cortical level in both hemispheres (Cheung et al., 2009, 2017; Kral et al., 2013). Furthermore, previous studies have demonstrated this development of an aural preference in SSD patients is increased when there is a longer duration of time in a state of asymmetric hearing (Polonenko et al., 2017).

Following CI, bimodal integration between ears also relies on central processes that may be impacted by duration of auditory deprivation (Balkenhol et al., 2020). Despite these centrally-mediated adaptations, however, our results suggest that in aggregate, SSD individuals implanted with 5-10-year duration of deafness still perform as well as those implanted earlier. On an individual level, 1 subject with 10-year duration of deafness did not experience any CNC benefit and there was also an emerging trend of subjects with longer duration of deafness experiencing a slower trajectory to plateau performance. This difference did not reach statistical significance, potentially due to inadequate statistical power as a result of a limited sample in our 5–10 year duration of deafness cohort. However, our findings are consistent with studies demonstrating longer durations of deafness being associated with decline in centrally-mediated adaptive processes that may result in a slower trajectory of auditory learning (Kral and Eggermont, 2007; Balkenhol et al., 2020). In pediatric populations with early- and even late-onset SSD, EEG activity has demonstrated a partial reversal of the cortical reorganization initiated at the onset of their hearing loss associated with consistent and chronic CI use (Lee et al., 2020). Collectively, this implicates the eventual benefits of CI in partially restoring the aural balance between ears impacted by SSD in adult patients as well, even if it may take longer in patients with longer durations of deafness.

### Speech understanding in noise and spatial hearing

In this study, head shadow (S_SSD_N_NH_), squelch (S_NH_N_SSD_), and summation (S_0_N_0_) effects were separately assessed in SSD CI patients (Figure 3). Consistent with previous studies (Buechner et al., 2010; Arndt et al., 2017), our data demonstrate SSD was most detrimental in the S_SSD_N_NH_ condition and CI improved patient performance by 6 months post-activation, driven partly by benefits associated with the HSE. In other spatial configurations, measured Az Bio scores demonstrated a ceiling effect, defined primarily by initial scores above 85% (Spahr et al., 2014), which may mask subsequent changes due to CI. This ceiling effect also has been found in prior studies and therefore, although AzBio is commonly used in the assessment of hearing in noise in bilateral hearing loss patients, the SSD population may require unique and more specific audiological testing to elucidate the true effect of CI on speech understanding in noise when the contralateral ear is normal (Litovsky et al., 2006; Massa and Ruckenstein, 2014).

While duration of deafness did not significantly impact speech understanding in noise (Figure 3) or patient-reported spatial hearing experience post-CI (Figure 4), we found that subjects with between 5 and 10-years duration of deafness tended to report less impact on spatial hearing experience prior to implantation compared to subjects with <5-year duration of deafness (Figure 4). This finding is consistent with central compensatory mechanisms that occur after monaural hearing loss. Studies have demonstrated that these mechanisms may lead to improvements in sound localization despite monaural hearing and lack of ILD and ITD cues. For instance, Agterberg et al. (2014) found that some SSD listeners are able to compensate for the loss of ILDs and ITDs by adopting high-frequency spectral-shape cues provided by the pinna of the hearing ear for sound localization in the horizontal plane, and that this ability deteriorates when the pinna is filled with a mold or if the hearing ear also has high-frequency hearing loss. Similar to our data, the authors also found large inter-subject variability in the extent of compensation. The SHQ survey used in our study also presents spatial hearing scenarios that predominantly relate to sound localization in the horizontal plane (e.g., direction of moving car, location of a talker in a room, etc.).

Our data are limited by the lack of objective sound localization testing, which have been previously studied in the SSD-CI population (Firszt et al., 2012; Gartrell et al., 2014; Ludwig et al., 2021). For instance, Litovsky et al. (2019) tested 9 SSD subjects with varying durations of deafness and did not identify overall differences in root-mean-square (RMS) errors between those with shorter vs. longer durations of deafness. However, subjects with longer durations of deafness were not tested with moving sound sources (e.g., direction of moving vehicle) that constitute important real-life scenarios presented in the SHQ. Overall, practical limitations have constrained objective sound localization testing to small series of research participants, unlike the large cohort reported in this study. While some studies (Heo et al., 2013; Ramakers et al., 2017) have demonstrated a significant correlation between objective measures and self-reported questionnaires assessing sound localization, further investigation is needed to define the extent to which objective sound localization testing concord with subjective real-world experience scored on survey instruments such as the SHQ. However, as CI is ultimately intended to improve the quality of life of individuals, it remains important to understand the real-world impact of SSD-CI and the extent to which central processes may play a role. Our data suggest that compensation may occur by 5 years of auditory deprivation in SSD subjects and can lead to improved real-world spatial hearing experience that consequently reduces the magnitude of perceived benefit related to spatial hearing after CI, despite objective gains in hearing in noise ability.

### Health utility

While previous studies have investigated quality of life changes related to SSD-CI, to our knowledge this is one of the first studies to measure health utility (Daher et al., 2023). HUI3 is a validated health utility instrument that allows not only comparison to other disease states but also downstream calculation of quality adjusted life years and cost-effectiveness, which are important measures for future health economic studies. Additionally, existing studies involving CI patients with bilateral hearing loss have already established that HUI3 is sensitive to hearing-related interventions (Kitterick et al., 2015). Though it is well-documented that hearing loss, including bilateral hearing loss, can have debilitating impact on quality of life, this is one of the first studies that quantifies the extent of this impact in SSD (Huddle et al., 2017; Nordvik et al., 2018; McRackan et al., 2019). Notably, when compared to other major conditions such as asthma, COPD, diabetes, and hypertension, SSD is associated with lower HUI3 scores prior to treatment, with scores comparable to that of stroke (Figure 5E). Considering that differences of 0.03 in HUI3 scores are regarded as clinically important (Samsa et al., 1999; Grootendorst et al., 2000; Drummond, 2001), this study highlights the profound impact of unilateral hearing loss that tends to be under-recognized.

Importantly, subdomain analysis of HUI3 scores shows that the reduced scores observed in SSD individuals prior to CI are primarily driven by the hearing subdomain (Figure 5B). This contrasts with other inner ear conditions where scores in the pain and emotion subdomains, which are indirectly related to the measured health condition and could be mediated by comorbid conditions such as depression and anxiety, are important drivers of poor health utility (Sun et al., 2014). CI use was associated with significant improvements in HUI3 scores over time (Figure 5A), suggesting an improvement in health-related quality of life consistent with previous studies (Muigg et al., 2020; Lindquist et al., 2023). Interestingly, greater variance in reported scores was observed at 6- and 12- months post activation in subjects with 5–10 years duration of deafness. This suggests there may be other factors contributing to this variability in CI experience that may be unmasked with longer periods of deafness and require further study. Interpretation of survey data is limited by both selection and reporting bias, as well as the lack of survey instruments specific to CI such as the CIQOL (McRackan et al., 2019). Nonetheless, taken together with audiological assessments and other patient-centered data, a more comprehensive picture on the real-world impact of SSD and subsequent electrical stimulation of the cochlea is obtained.

### Device use

Device use data provides us with information regarding the real-world usage of subject CIs. Device use was similar regardless of duration of deafness and remained stable through 12-months post-CI activation (Figure 6). Further, in our data, increased device use is associated with higher CNC scores (Supplementary Table 5). While this has also been reported in previous studies, the direction of causality remains to be confirmed as it is possible that subjects are reducing their device use when there is insufficient benefit, rather than poor performance solely due to lack of use (Holder et al., 2020; Holder and Gifford, 2021).

Taken together, our findings demonstrate the debilitating impact of SSD and the benefits of CI in individuals with short- and medium-term auditory deprivation, though some trends in the data may suggest differential impacts of CI may arise after 5 years of auditory deprivation. Centrally-mediated processes involving compensation after SSD and adaptation to bimodal hearing after CI are understood to play important roles in auditory processing after SSD-CI. Data presented here illustrate the changes in audition related to those processes and their correlation to individual experiences in real-world settings.

## Limitations

For this prospective observational study, intrinsic limitations exist related to missing data and statistical power, particularly in the cohort with longer duration of auditory deprivation. Although drop-out occurred in only 1 subject over the course of the study, survey response rates were more variable and vulnerable to reporting and selection biases. Further, sound localization testing was not performed due to practical limitations associated with the size of the study. Additionally, both objective and subjective assessments for CI subjects are imperfect and the instruments used in our study reflect both the limitations associated with those assessments and opportunities for instrument development specifically for the SSD population. Several confounding variables could have influenced some of our findings; as such, further studies in addition to our preliminary analysis of duration of deafness are encouraged to explore the effects of such variables, such as hearing aid use prior to implantation and surgical factors at the point of implantation. Though the literature is sparse, some evidence exists in adult listeners of varying hearing impairments to suggest a positive association (*r* ~ 0.3) between cognitive function and speech perception in noise (Dryden et al., 2017). As such, we encourage additional cognitive function testing in the SSD-CI population to help elucidate the impact SSD may have on cognition and subsequently demonstrate any secondary effects on speech perception performance. Further studies comparing the effects of CI in subjects with unilateral hearing loss and in subjects with bilateral hearing loss may also be needed to understand the extent to which CI restores bilateral hearing and benefits patients as a treatment option for these conditions. Lastly, further research is needed to fully capture the neural mechanisms that may be responsible for auditory adaptations in individuals with SSD, especially in those with longer durations of deafness.

## Data availability statement

The raw data supporting the conclusions of this article will be made available by the authors, without undue reservation.

## Ethics statement

The studies involving humans were approved by Johns Hopkins Medicine Institutional Review Boards. The studies were conducted in accordance with the local legislation and institutional requirements. The participants provided their written informed consent to participate in this study.

## Author contributions

MU and DS have seen the original study data, reviewed the analysis of the data, and wrote and approved the final manuscript. SB has seen the original study data, reviewed the analysis of the data, and approved the final manuscript. SS reviewed the analysis of the data and approved the final manuscript. AC, CC, RD, DM, and JY assisted in implementation and data collection, and approved the final manuscript. All authors contributed to the article and approved the submitted version.

## Funding

This work was supported in part by the NIDCD Grant No. 5T32DC000027-33.
