# Supplementary material for: Cochlear implantation in unilateral hearing loss: impact of short- to medium-term auditory deprivation
Source: Front Neurosci. 2023 Oct 9;17:1247269. doi: 10.3389/fnins.2023.1247269 (PMC10591100; doi:10.3389/fnins.2023.1247269)
Supplement: Supplementary file 1 [file Presentation_1.PPTX]

## Slide 1
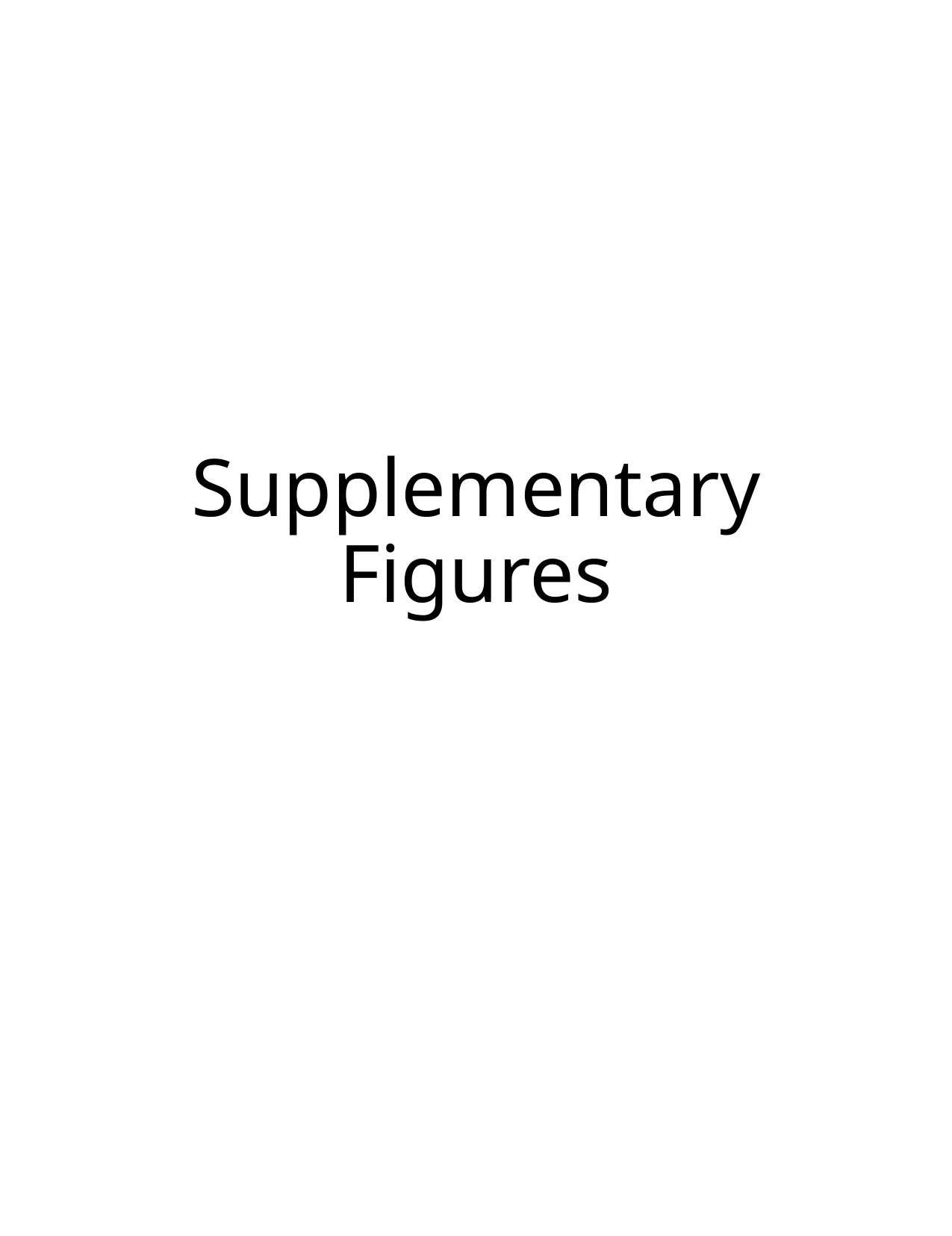

# Supplementary Figures

## Slide 2
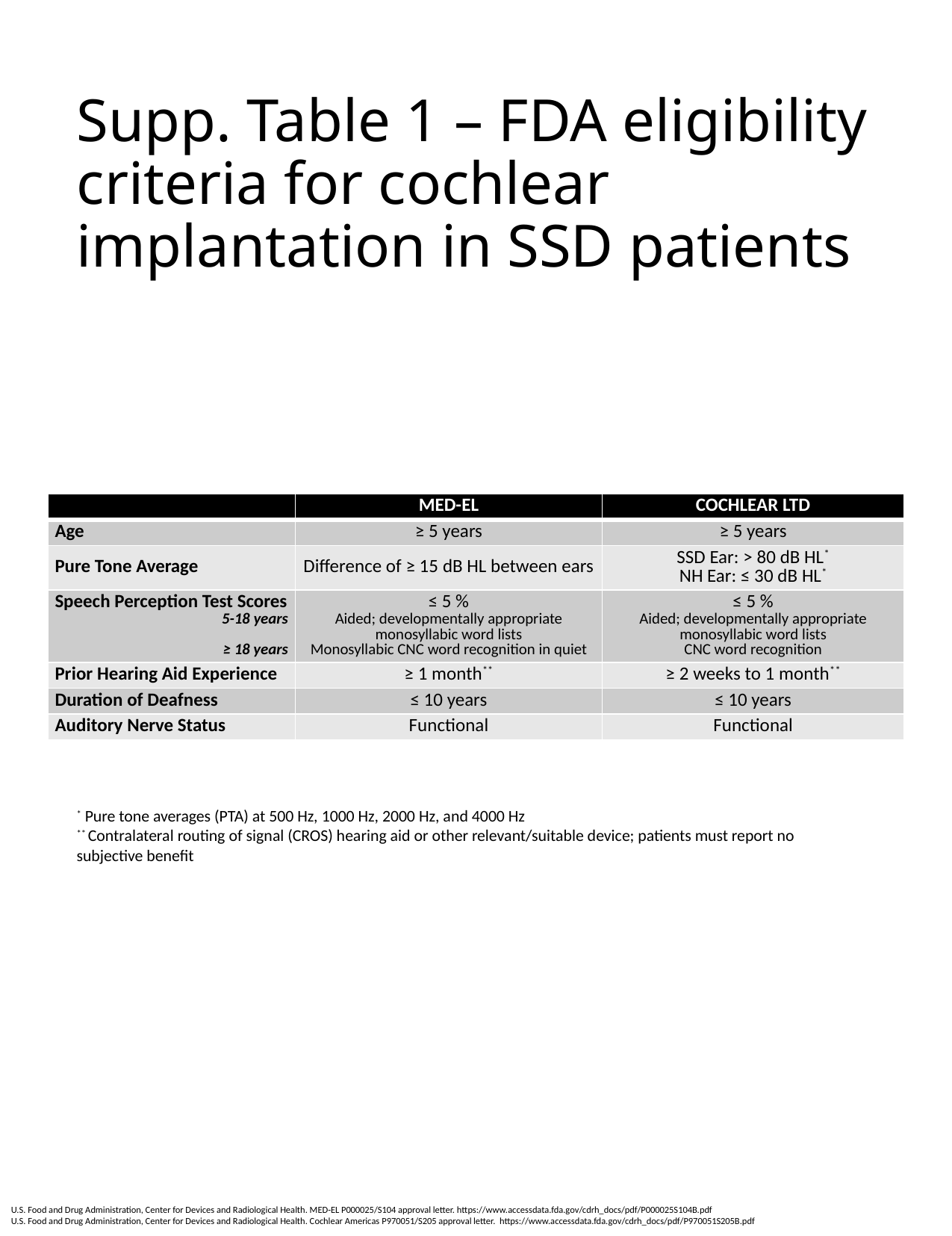

# Supp. Table 1 – FDA eligibility criteria for cochlear implantation in SSD patients
| | MED-EL | COCHLEAR LTD |
| --- | --- | --- |
| Age | ≥ 5 years | ≥ 5 years |
| Pure Tone Average | Difference of ≥ 15 dB HL between ears | SSD Ear: > 80 dB HL\* NH Ear: ≤ 30 dB HL\* |
| Speech Perception Test Scores 5-18 years ≥ 18 years | ≤ 5 % Aided; developmentally appropriate monosyllabic word lists Monosyllabic CNC word recognition in quiet | ≤ 5 % Aided; developmentally appropriate monosyllabic word lists CNC word recognition |
| Prior Hearing Aid Experience | ≥ 1 month\*\* | ≥ 2 weeks to 1 month\*\* |
| Duration of Deafness | ≤ 10 years | ≤ 10 years |
| Auditory Nerve Status | Functional | Functional |
* Pure tone averages (PTA) at 500 Hz, 1000 Hz, 2000 Hz, and 4000 Hz
** Contralateral routing of signal (CROS) hearing aid or other relevant/suitable device; patients must report no subjective benefit
U.S. Food and Drug Administration, Center for Devices and Radiological Health. MED-EL P000025/S104 approval letter. https://www.accessdata.fda.gov/cdrh_docs/pdf/P000025S104B.pdf
U.S. Food and Drug Administration, Center for Devices and Radiological Health. Cochlear Americas P970051/S205 approval letter. https://www.accessdata.fda.gov/cdrh_docs/pdf/P970051S205B.pdf

## Slide 3
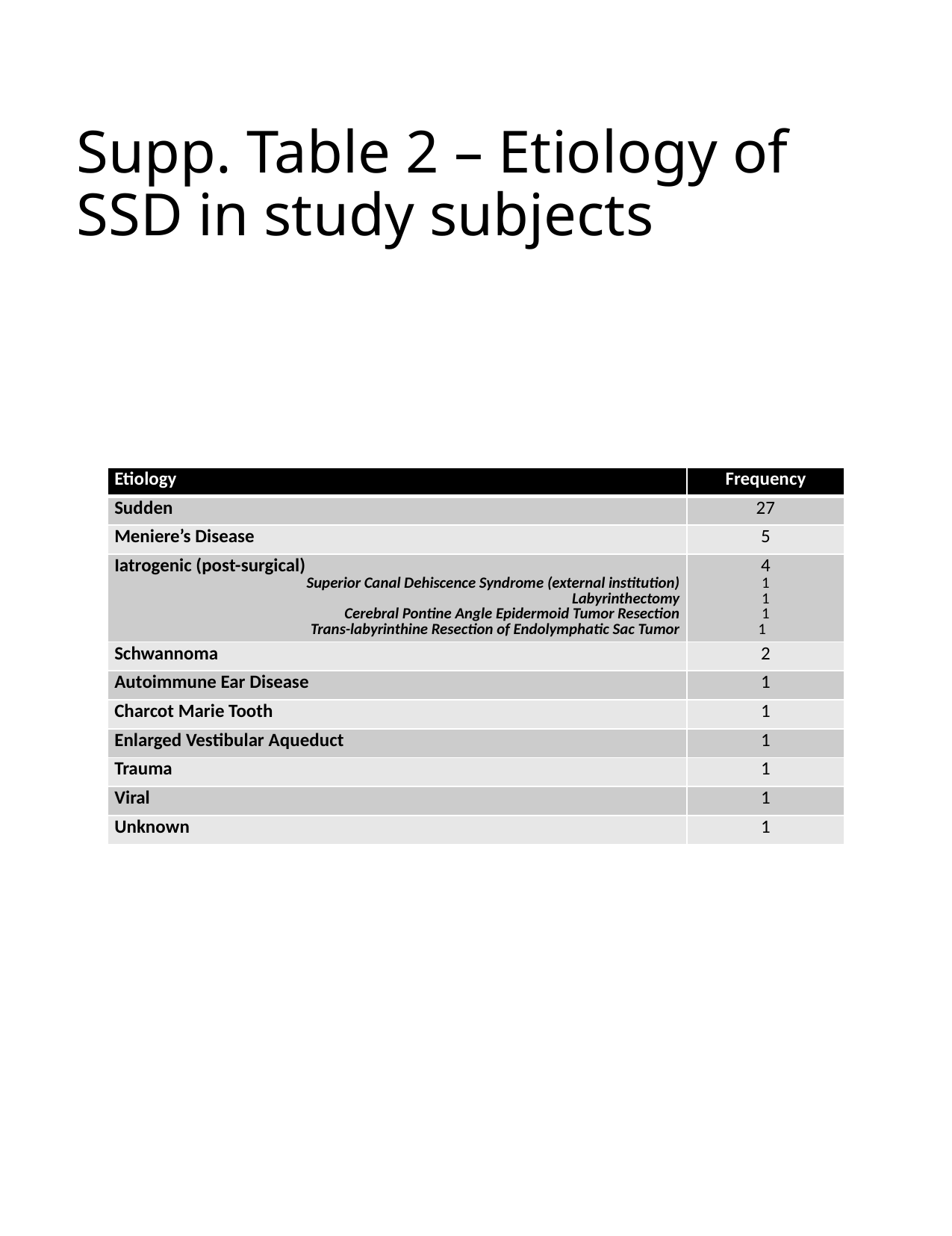

# Supp. Table 2 – Etiology of SSD in study subjects
| Etiology | Frequency |
| --- | --- |
| Sudden | 27 |
| Meniere’s Disease | 5 |
| Iatrogenic (post-surgical) Superior Canal Dehiscence Syndrome (external institution) Labyrinthectomy Cerebral Pontine Angle Epidermoid Tumor Resection Trans-labyrinthine Resection of Endolymphatic Sac Tumor | 4 1 1 1 1 |
| Schwannoma | 2 |
| Autoimmune Ear Disease | 1 |
| Charcot Marie Tooth | 1 |
| Enlarged Vestibular Aqueduct | 1 |
| Trauma | 1 |
| Viral | 1 |
| Unknown | 1 |

## Slide 4
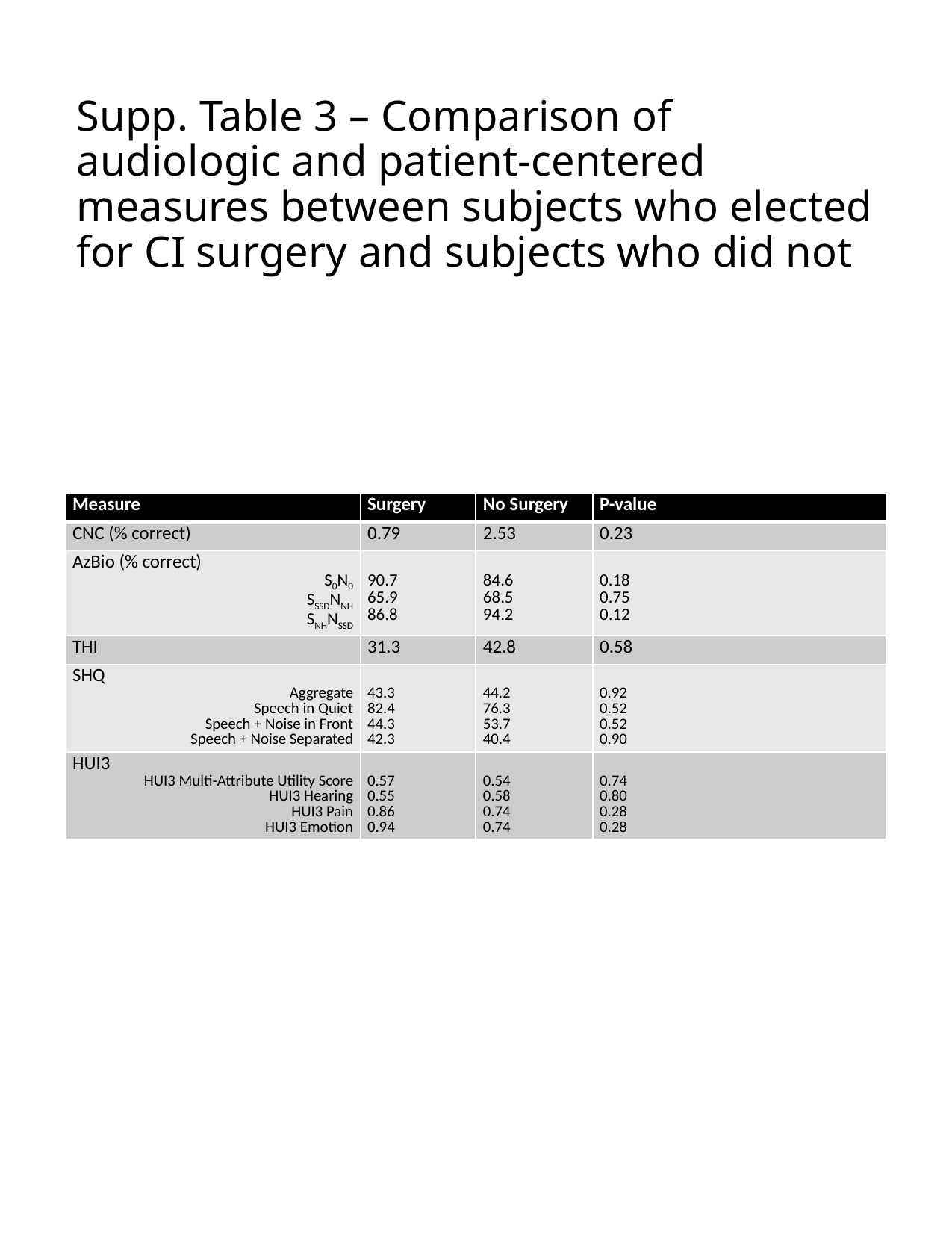

# Supp. Table 3 – Comparison of audiologic and patient-centered measures between subjects who elected for CI surgery and subjects who did not
| Measure | Surgery | No Surgery | P-value |
| --- | --- | --- | --- |
| CNC (% correct) | 0.79 | 2.53 | 0.23 |
| AzBio (% correct) S0N0 SSSDNNH SNHNSSD | 90.7 65.9 86.8 | 84.6 68.5 94.2 | 0.18 0.75 0.12 |
| THI | 31.3 | 42.8 | 0.58 |
| SHQ Aggregate Speech in Quiet Speech + Noise in Front Speech + Noise Separated | 43.3 82.4 44.3 42.3 | 44.2 76.3 53.7 40.4 | 0.92 0.52 0.52 0.90 |
| HUI3 HUI3 Multi-Attribute Utility Score HUI3 Hearing HUI3 Pain HUI3 Emotion | 0.57 0.55 0.86 0.94 | 0.54 0.58 0.74 0.74 | 0.74 0.80 0.28 0.28 |

## Slide 5
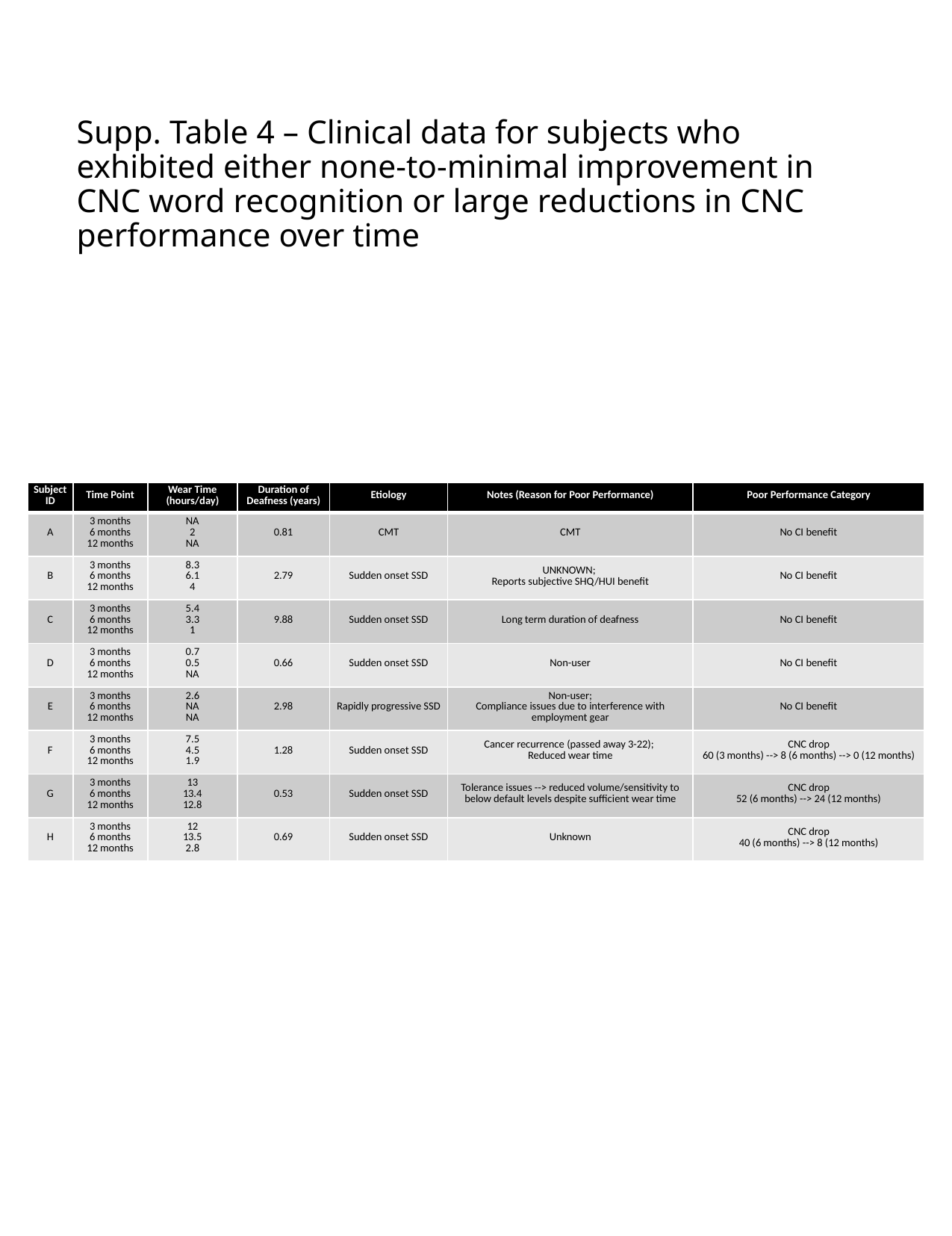

# Supp. Table 4 – Clinical data for subjects who exhibited either none-to-minimal improvement in CNC word recognition or large reductions in CNC performance over time
| Subject ID | Time Point | Wear Time (hours/day) | Duration of Deafness (years) | Etiology | Notes (Reason for Poor Performance) | Poor Performance Category |
| --- | --- | --- | --- | --- | --- | --- |
| A | 3 months 6 months 12 months | NA 2 NA | 0.81 | CMT | CMT | No CI benefit |
| B | 3 months 6 months 12 months | 8.3 6.1 4 | 2.79 | Sudden onset SSD | UNKNOWN; Reports subjective SHQ/HUI benefit | No CI benefit |
| C | 3 months 6 months 12 months | 5.4 3.3 1 | 9.88 | Sudden onset SSD | Long term duration of deafness | No CI benefit |
| D | 3 months 6 months 12 months | 0.7 0.5 NA | 0.66 | Sudden onset SSD | Non-user | No CI benefit |
| E | 3 months 6 months 12 months | 2.6 NA NA | 2.98 | Rapidly progressive SSD | Non-user; Compliance issues due to interference with employment gear | No CI benefit |
| F | 3 months 6 months 12 months | 7.5 4.5 1.9 | 1.28 | Sudden onset SSD | Cancer recurrence (passed away 3-22); Reduced wear time | CNC drop 60 (3 months) --> 8 (6 months) --> 0 (12 months) |
| G | 3 months 6 months 12 months | 13 13.4 12.8 | 0.53 | Sudden onset SSD | Tolerance issues --> reduced volume/sensitivity to below default levels despite sufficient wear time | CNC drop 52 (6 months) --> 24 (12 months) |
| H | 3 months 6 months 12 months | 12 13.5 2.8 | 0.69 | Sudden onset SSD | Unknown | CNC drop 40 (6 months) --> 8 (12 months) |

## Slide 6
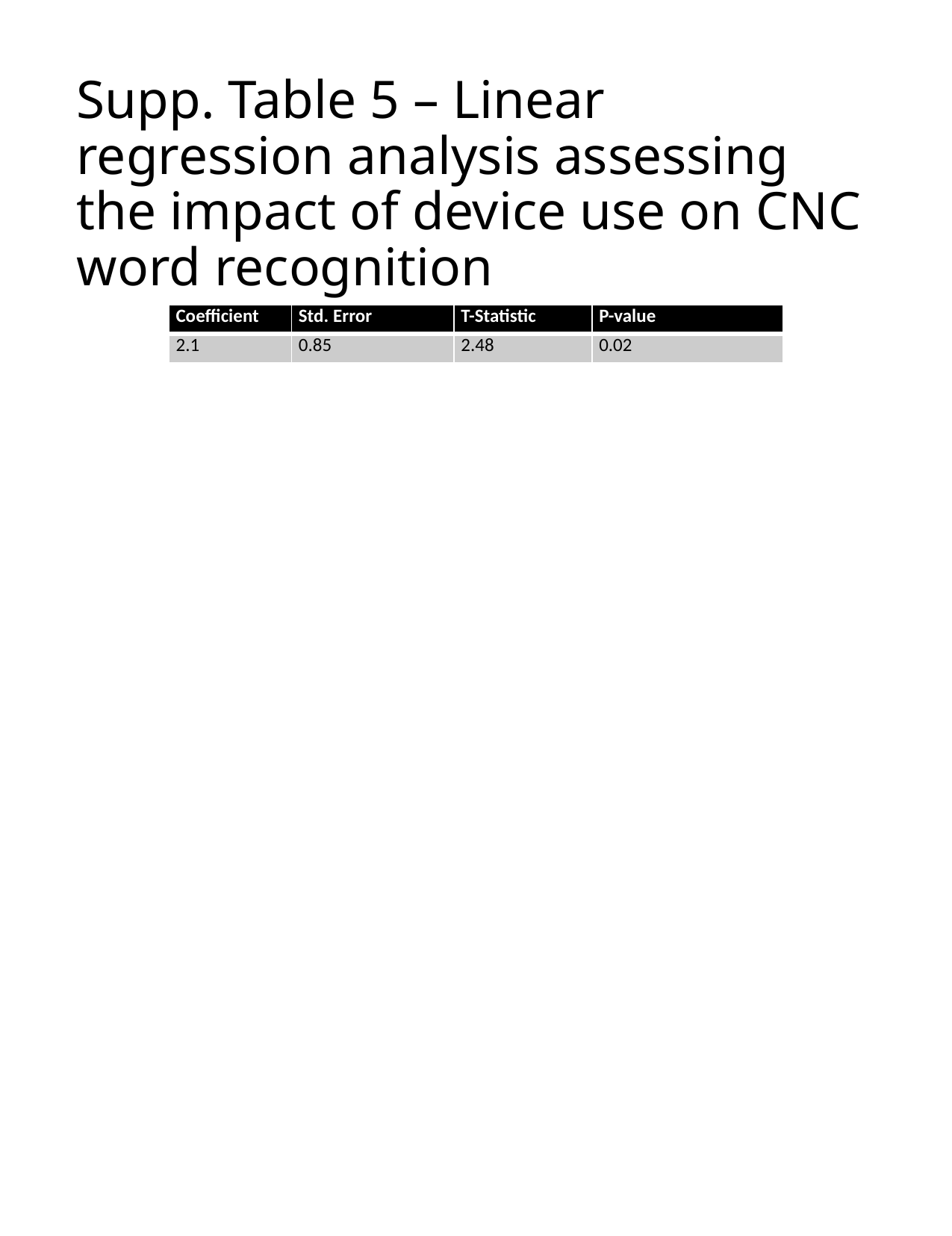

# Supp. Table 5 – Linear regression analysis assessing the impact of device use on CNC word recognition
| Coefficient | Std. Error | T-Statistic | P-value |
| --- | --- | --- | --- |
| 2.1 | 0.85 | 2.48 | 0.02 |

## Slide 7
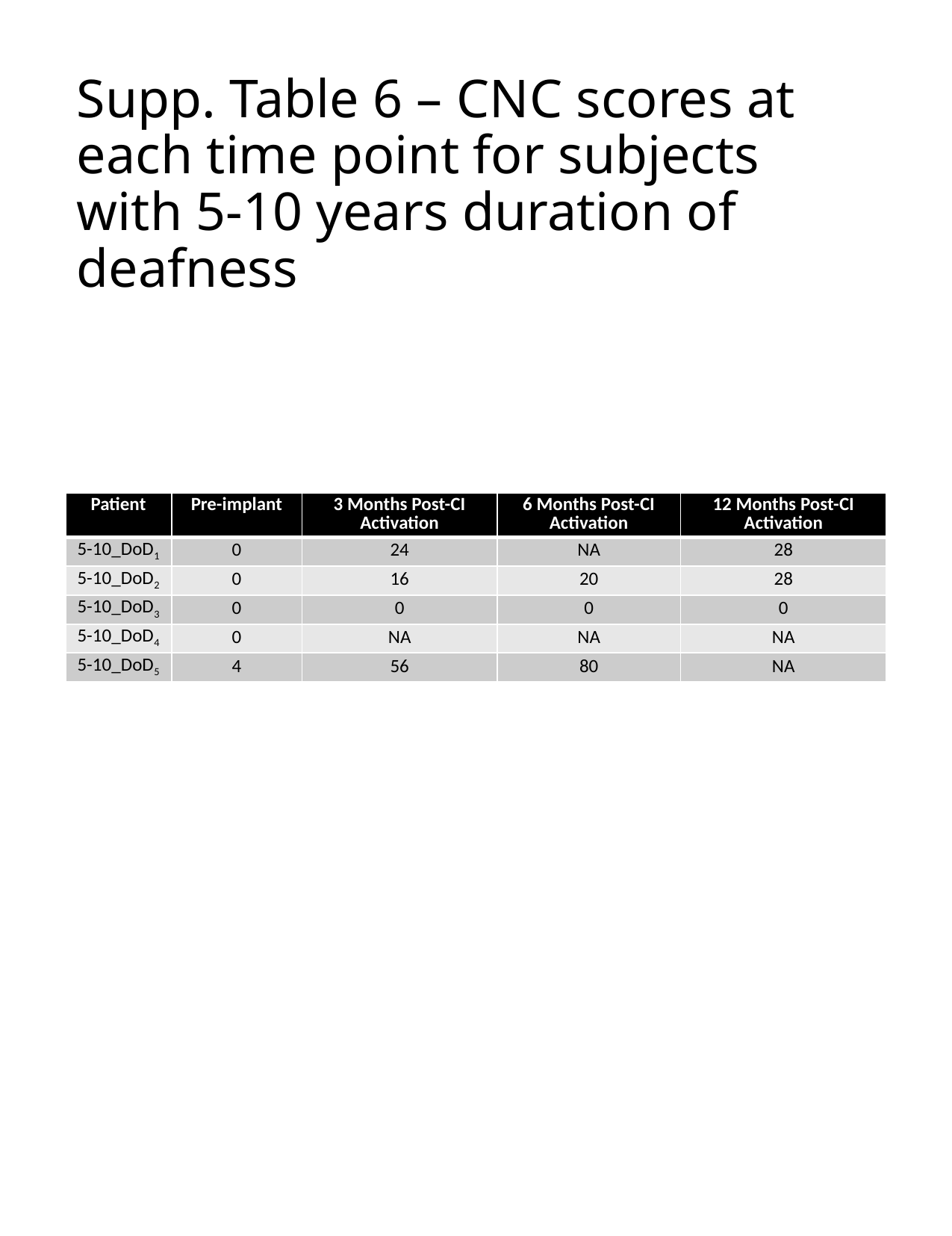

# Supp. Table 6 – CNC scores at each time point for subjects with 5-10 years duration of deafness
| Patient | Pre-implant | 3 Months Post-CI Activation | 6 Months Post-CI Activation | 12 Months Post-CI Activation |
| --- | --- | --- | --- | --- |
| 5-10\_DoD1 | 0 | 24 | NA | 28 |
| 5-10\_DoD2 | 0 | 16 | 20 | 28 |
| 5-10\_DoD3 | 0 | 0 | 0 | 0 |
| 5-10\_DoD4 | 0 | NA | NA | NA |
| 5-10\_DoD5 | 4 | 56 | 80 | NA |

## Slide 8
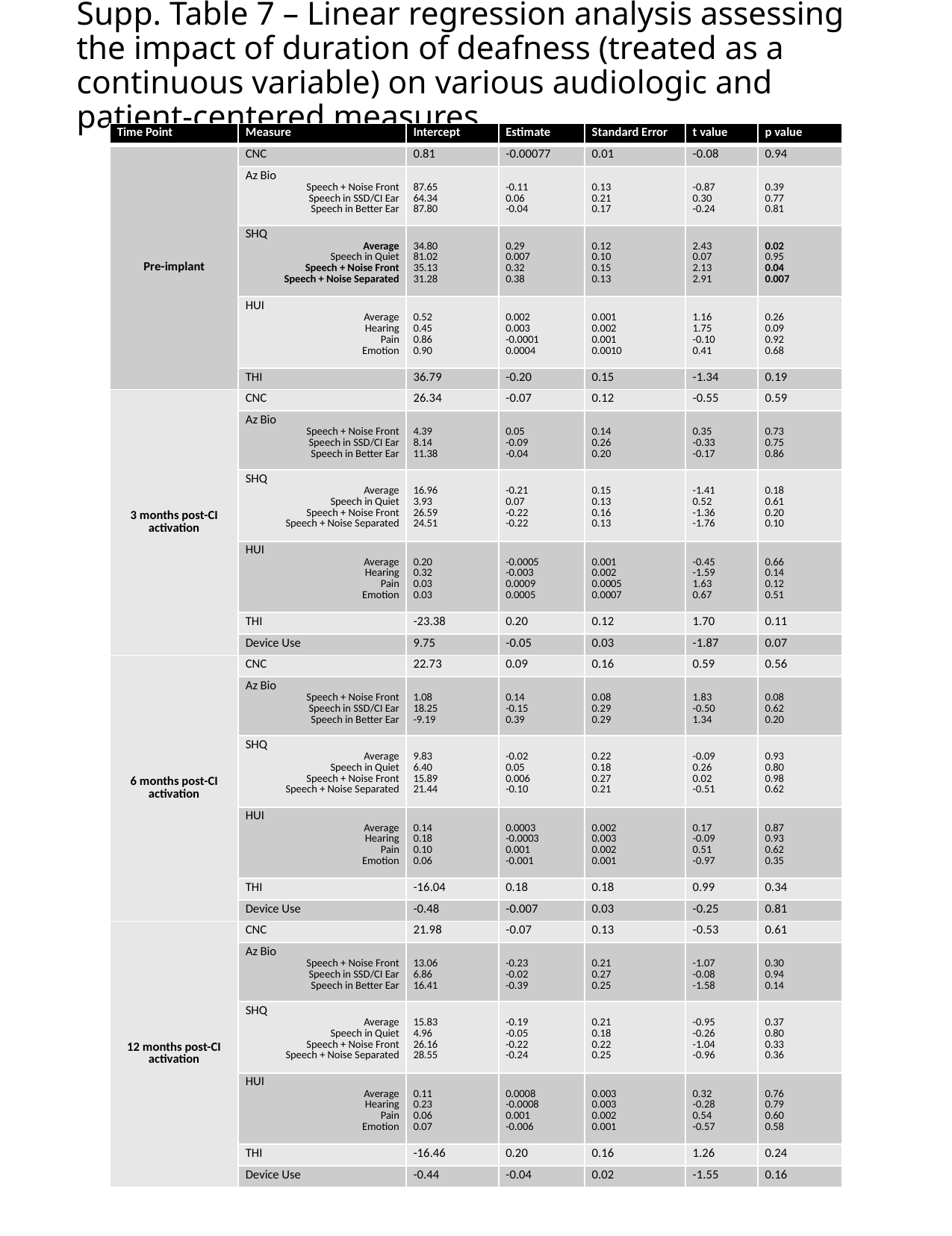

# Supp. Table 7 – Linear regression analysis assessing the impact of duration of deafness (treated as a continuous variable) on various audiologic and patient-centered measures
| Time Point | Measure | Intercept | Estimate | Standard Error | t value | p value |
| --- | --- | --- | --- | --- | --- | --- |
| Pre-implant | CNC | 0.81 | -0.00077 | 0.01 | -0.08 | 0.94 |
| | Az Bio Speech + Noise Front Speech in SSD/CI Ear Speech in Better Ear | 87.65 64.34 87.80 | -0.11 0.06 -0.04 | 0.13 0.21 0.17 | -0.87 0.30 -0.24 | 0.39 0.77 0.81 |
| | SHQ Average Speech in Quiet Speech + Noise Front Speech + Noise Separated | 34.80 81.02 35.13 31.28 | 0.29 0.007 0.32 0.38 | 0.12 0.10 0.15 0.13 | 2.43 0.07 2.13 2.91 | 0.02 0.95 0.04 0.007 |
| | HUI Average Hearing Pain Emotion | 0.52 0.45 0.86 0.90 | 0.002 0.003 -0.0001 0.0004 | 0.001 0.002 0.001 0.0010 | 1.16 1.75 -0.10 0.41 | 0.26 0.09 0.92 0.68 |
| | THI | 36.79 | -0.20 | 0.15 | -1.34 | 0.19 |
| 3 months post-CI activation | CNC | 26.34 | -0.07 | 0.12 | -0.55 | 0.59 |
| | Az Bio Speech + Noise Front Speech in SSD/CI Ear Speech in Better Ear | 4.39 8.14 11.38 | 0.05 -0.09 -0.04 | 0.14 0.26 0.20 | 0.35 -0.33 -0.17 | 0.73 0.75 0.86 |
| | SHQ Average Speech in Quiet Speech + Noise Front Speech + Noise Separated | 16.96 3.93 26.59 24.51 | -0.21 0.07 -0.22 -0.22 | 0.15 0.13 0.16 0.13 | -1.41 0.52 -1.36 -1.76 | 0.18 0.61 0.20 0.10 |
| | HUI Average Hearing Pain Emotion | 0.20 0.32 0.03 0.03 | -0.0005 -0.003 0.0009 0.0005 | 0.001 0.002 0.0005 0.0007 | -0.45 -1.59 1.63 0.67 | 0.66 0.14 0.12 0.51 |
| | THI | -23.38 | 0.20 | 0.12 | 1.70 | 0.11 |
| | Device Use | 9.75 | -0.05 | 0.03 | -1.87 | 0.07 |
| 6 months post-CI activation | CNC | 22.73 | 0.09 | 0.16 | 0.59 | 0.56 |
| | Az Bio Speech + Noise Front Speech in SSD/CI Ear Speech in Better Ear | 1.08 18.25 -9.19 | 0.14 -0.15 0.39 | 0.08 0.29 0.29 | 1.83 -0.50 1.34 | 0.08 0.62 0.20 |
| | SHQ Average Speech in Quiet Speech + Noise Front Speech + Noise Separated | 9.83 6.40 15.89 21.44 | -0.02 0.05 0.006 -0.10 | 0.22 0.18 0.27 0.21 | -0.09 0.26 0.02 -0.51 | 0.93 0.80 0.98 0.62 |
| | HUI Average Hearing Pain Emotion | 0.14 0.18 0.10 0.06 | 0.0003 -0.0003 0.001 -0.001 | 0.002 0.003 0.002 0.001 | 0.17 -0.09 0.51 -0.97 | 0.87 0.93 0.62 0.35 |
| | THI | -16.04 | 0.18 | 0.18 | 0.99 | 0.34 |
| | Device Use | -0.48 | -0.007 | 0.03 | -0.25 | 0.81 |
| 12 months post-CI activation | CNC | 21.98 | -0.07 | 0.13 | -0.53 | 0.61 |
| | Az Bio Speech + Noise Front Speech in SSD/CI Ear Speech in Better Ear | 13.06 6.86 16.41 | -0.23 -0.02 -0.39 | 0.21 0.27 0.25 | -1.07 -0.08 -1.58 | 0.30 0.94 0.14 |
| | SHQ Average Speech in Quiet Speech + Noise Front Speech + Noise Separated | 15.83 4.96 26.16 28.55 | -0.19 -0.05 -0.22 -0.24 | 0.21 0.18 0.22 0.25 | -0.95 -0.26 -1.04 -0.96 | 0.37 0.80 0.33 0.36 |
| | HUI Average Hearing Pain Emotion | 0.11 0.23 0.06 0.07 | 0.0008 -0.0008 0.001 -0.006 | 0.003 0.003 0.002 0.001 | 0.32 -0.28 0.54 -0.57 | 0.76 0.79 0.60 0.58 |
| | THI | -16.46 | 0.20 | 0.16 | 1.26 | 0.24 |
| | Device Use | -0.44 | -0.04 | 0.02 | -1.55 | 0.16 |

## Slide 9
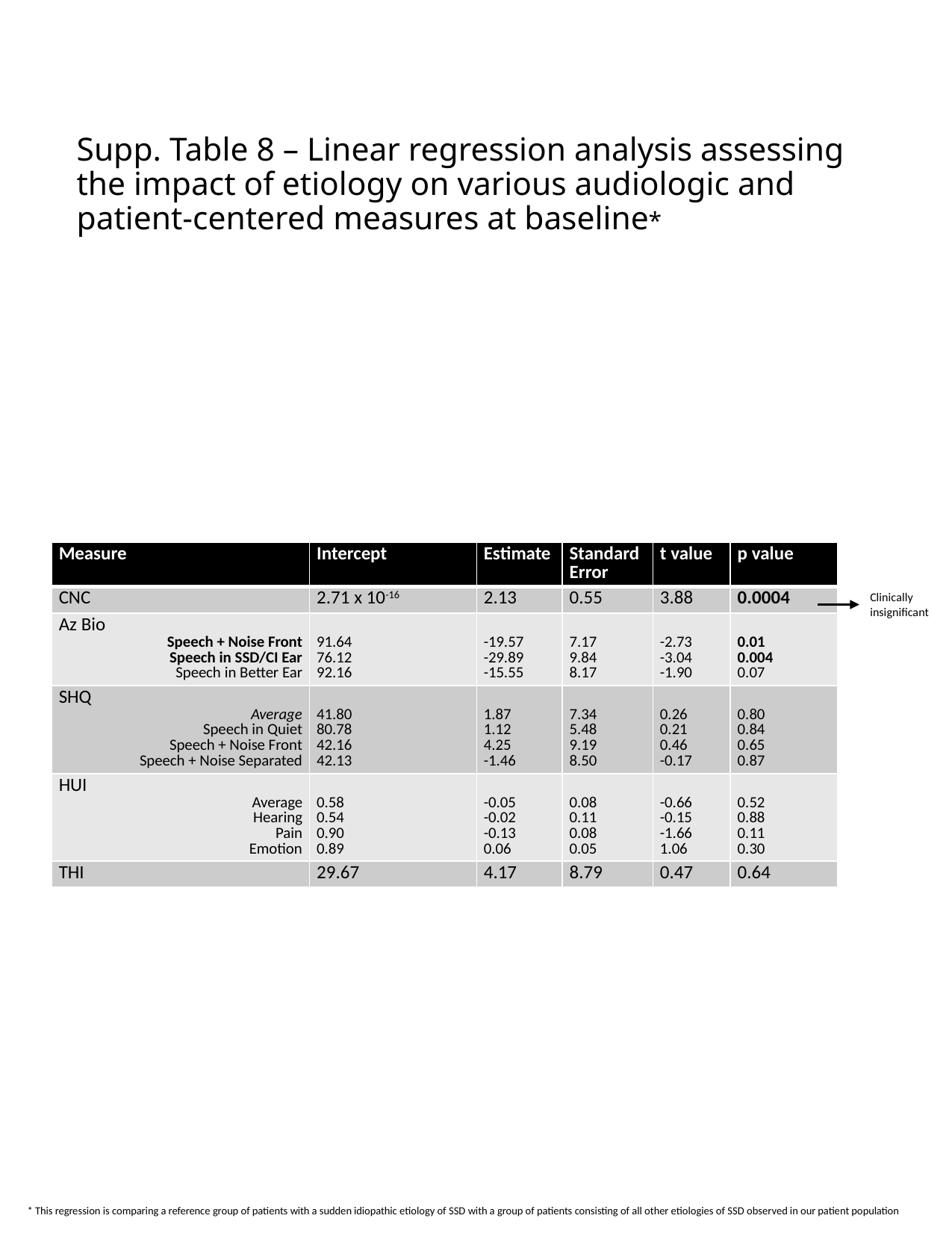

# Supp. Table 8 – Linear regression analysis assessing the impact of etiology on various audiologic and patient-centered measures at baseline*
| Measure | Intercept | Estimate | Standard Error | t value | p value |
| --- | --- | --- | --- | --- | --- |
| CNC | 2.71 x 10^-16^ | 2.13 | 0.55 | 3.88 | 0.0004 |
| Az Bio Speech + Noise Front Speech in SSD/CI Ear Speech in Better Ear | 91.64 76.12 92.16 | -19.57 -29.89 -15.55 | 7.17 9.84 8.17 | -2.73 -3.04 -1.90 | 0.01 0.004 0.07 |
| SHQ Average Speech in Quiet Speech + Noise Front Speech + Noise Separated | 41.80 80.78 42.16 42.13 | 1.87 1.12 4.25 -1.46 | 7.34 5.48 9.19 8.50 | 0.26 0.21 0.46 -0.17 | 0.80 0.84 0.65 0.87 |
| HUI Average Hearing Pain Emotion | 0.58 0.54 0.90 0.89 | -0.05 -0.02 -0.13 0.06 | 0.08 0.11 0.08 0.05 | -0.66 -0.15 -1.66 1.06 | 0.52 0.88 0.11 0.30 |
| THI | 29.67 | 4.17 | 8.79 | 0.47 | 0.64 |
Clinically
insignificant
* This regression is comparing a reference group of patients with a sudden idiopathic etiology of SSD with a group of patients consisting of all other etiologies of SSD observed in our patient population

## Slide 10
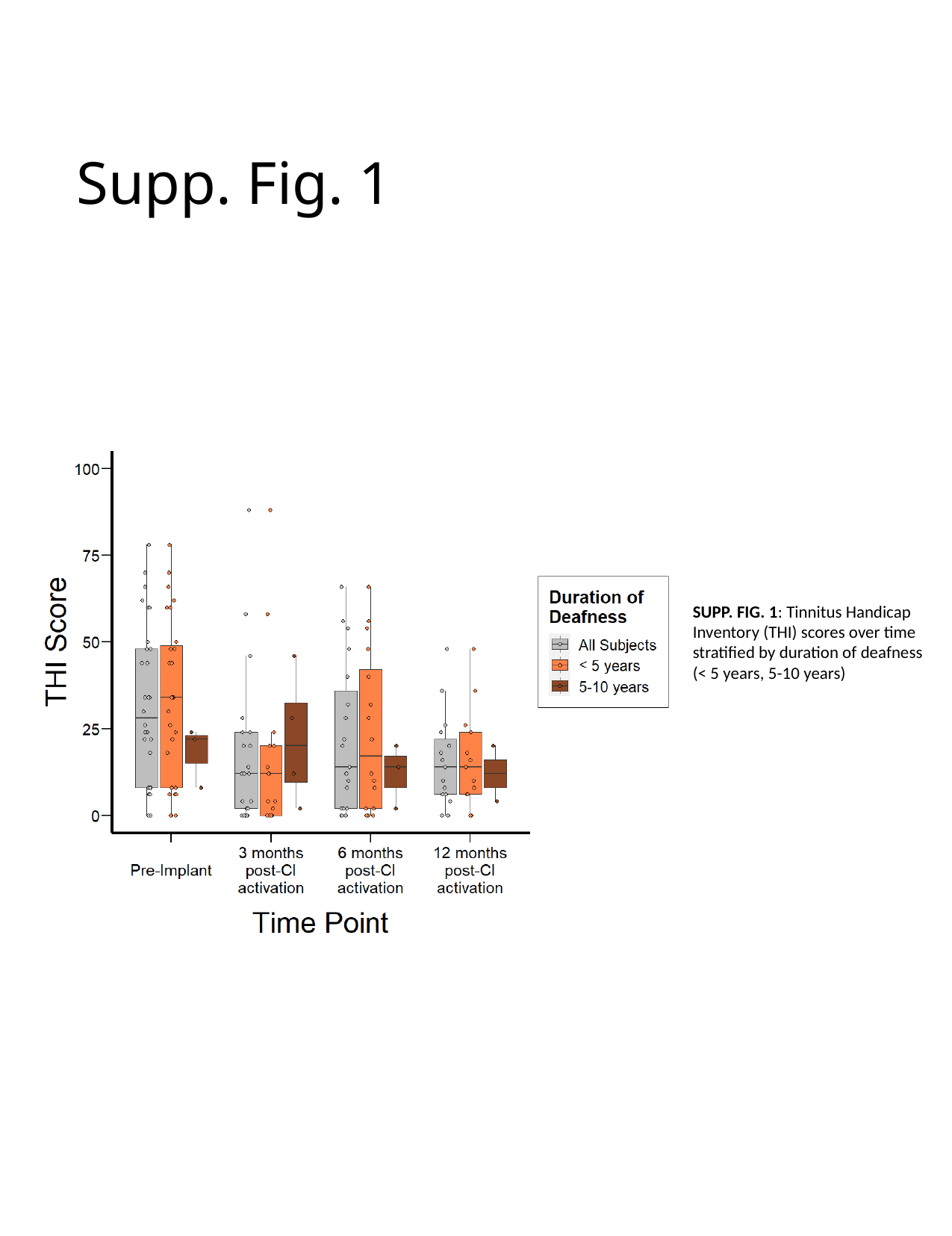

# Supp. Fig. 1
SUPP. FIG. 1: Tinnitus Handicap Inventory (THI) scores over time stratified by duration of deafness (< 5 years, 5-10 years)

## Slide 11
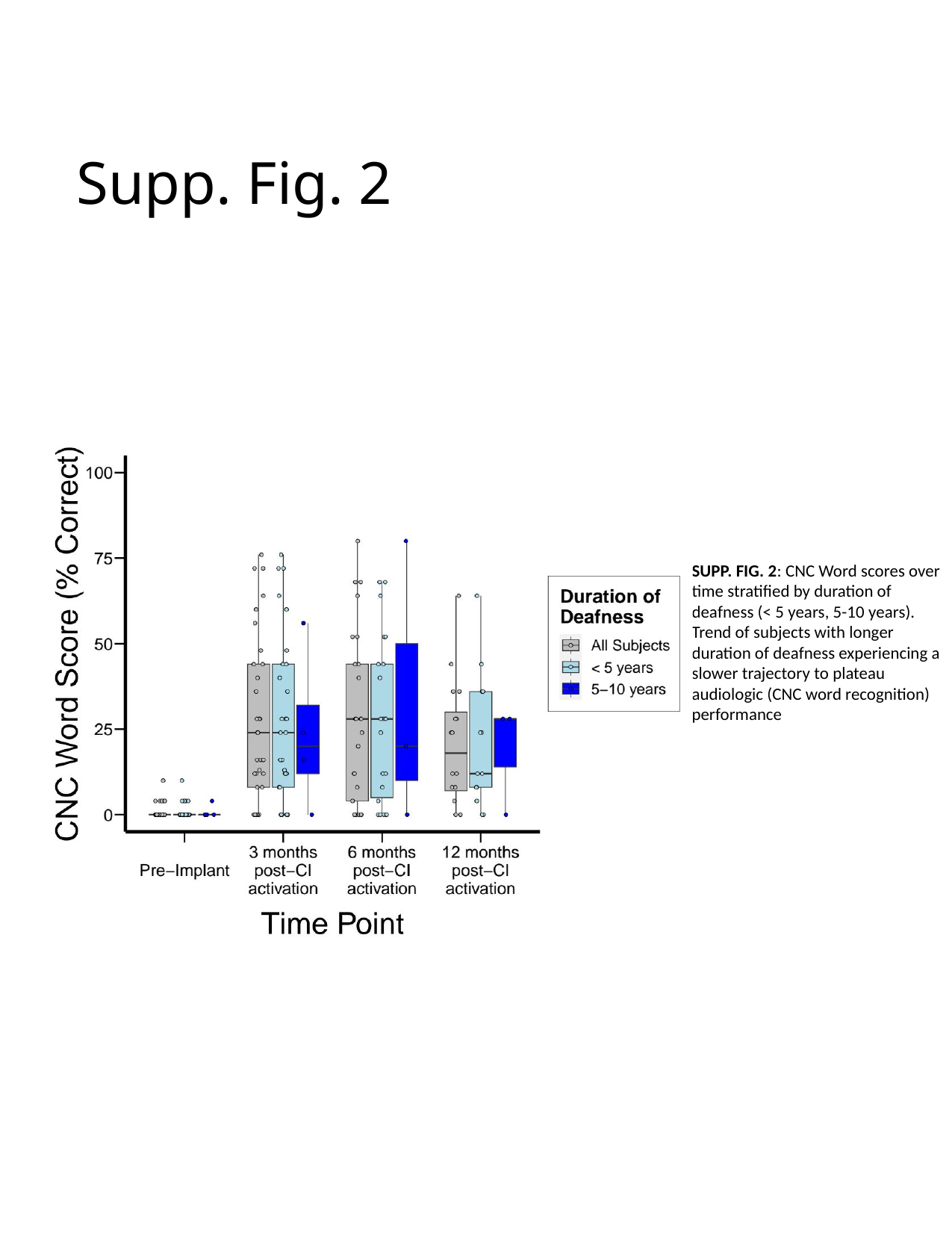

# Supp. Fig. 2
SUPP. FIG. 2: CNC Word scores over time stratified by duration of deafness (< 5 years, 5-10 years). Trend of subjects with longer duration of deafness experiencing a slower trajectory to plateau audiologic (CNC word recognition) performance
